# Supplementary figures and images for: A Warburg-like metabolic program coordinates Wnt, AMPK, and mTOR signaling pathways in epileptogenesis
Source: PLoS One. 2021 Aug 6;16(8):e0252282. doi: 10.1371/journal.pone.0252282 (PMC8345866; doi:10.1371/journal.pone.0252282)

A.

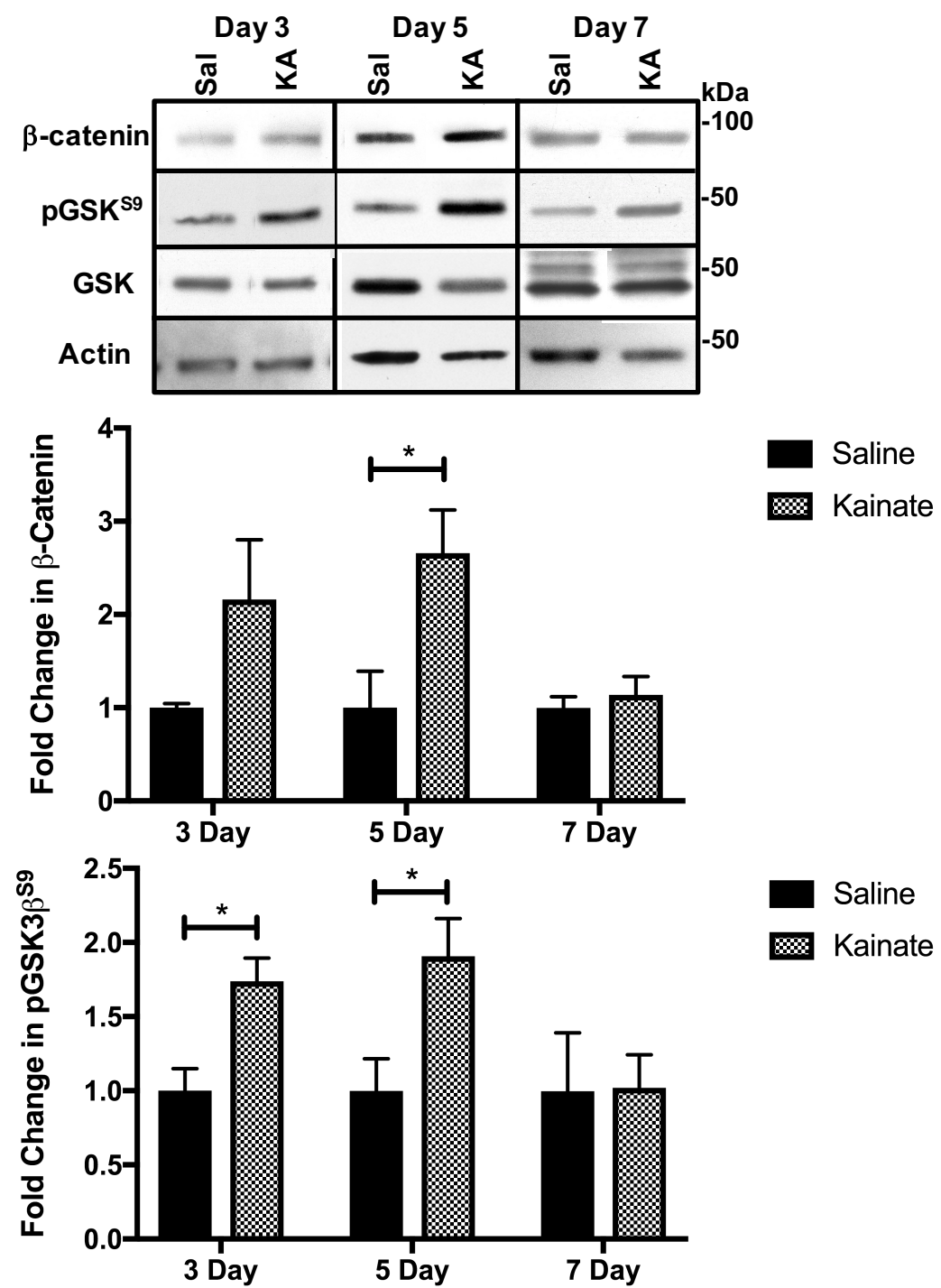

B.

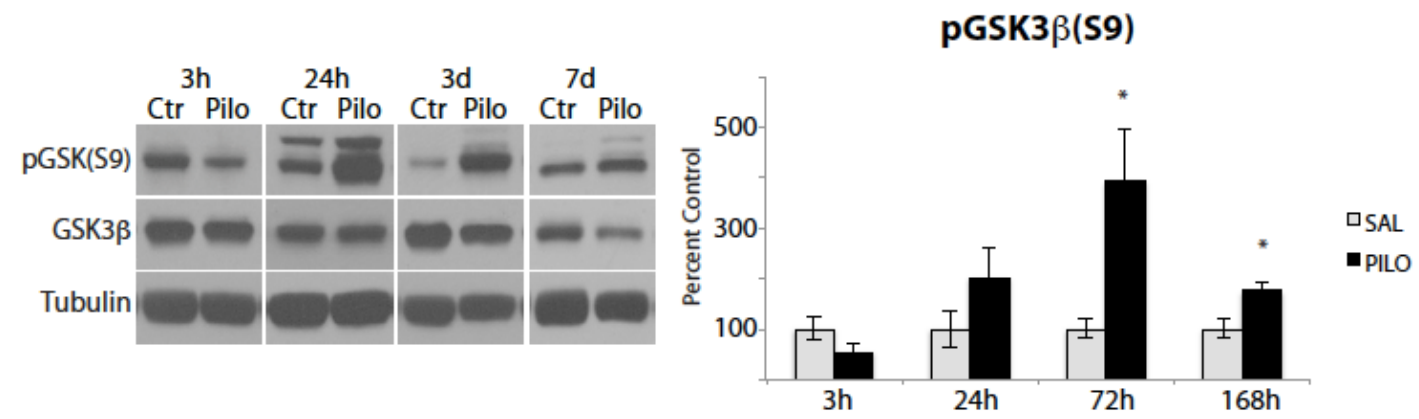

C.

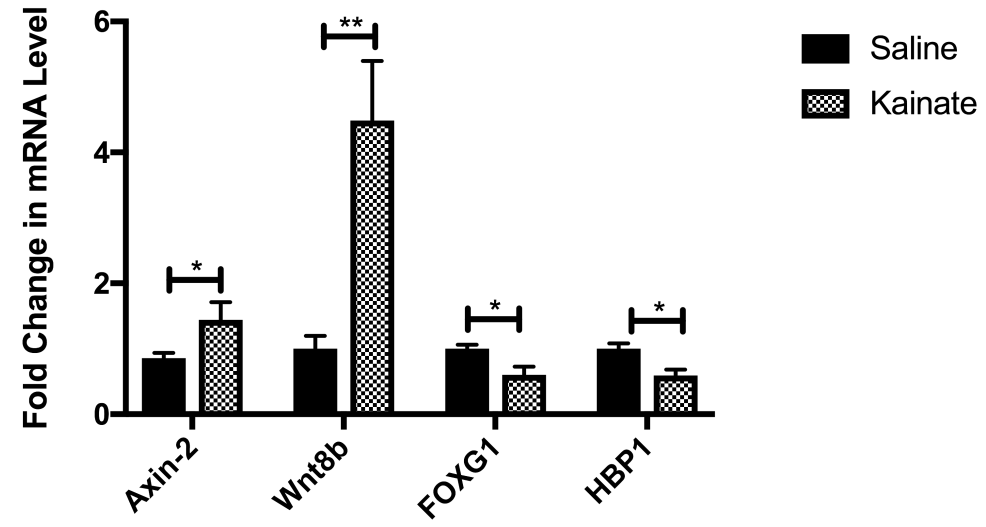

D.

Saline

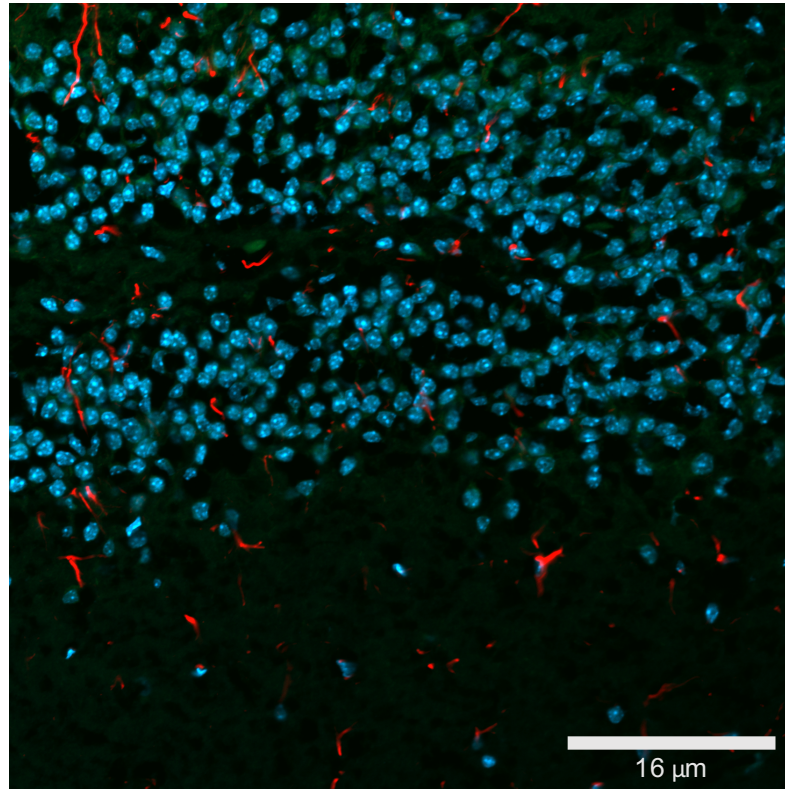

Pilocarpine

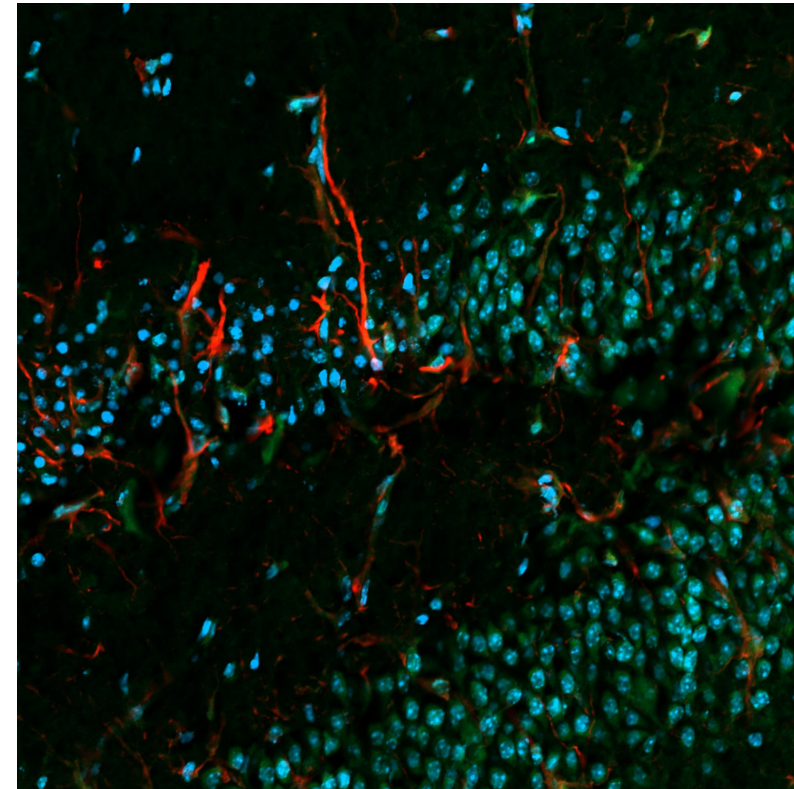

GFAP pGSK3 $\beta$ <sup>S9</sup> DAPI

E.

Saline

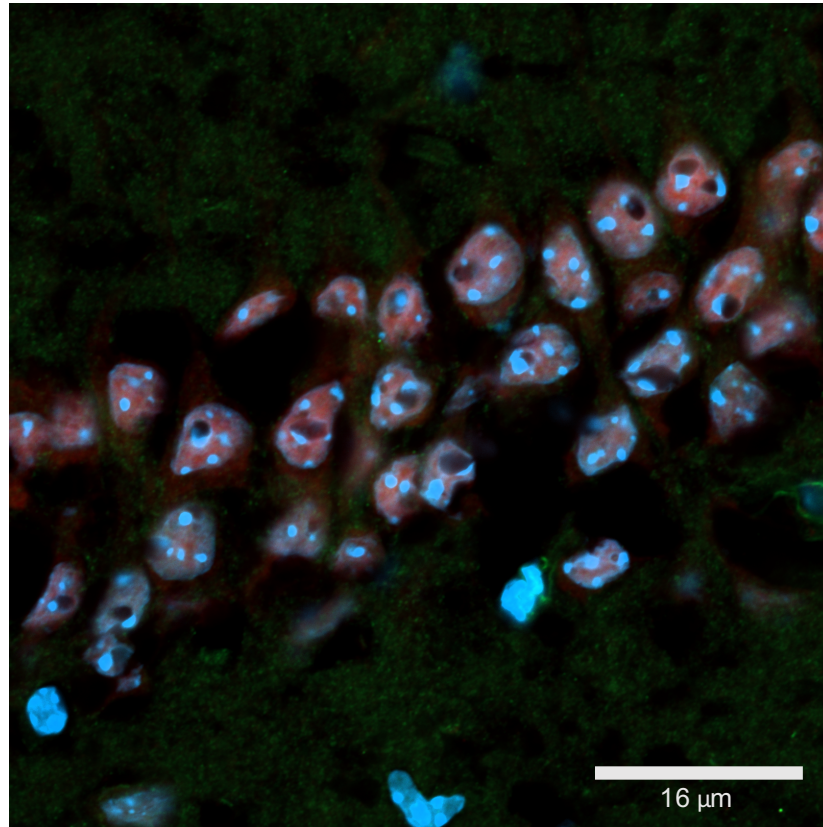

Pilocarpine

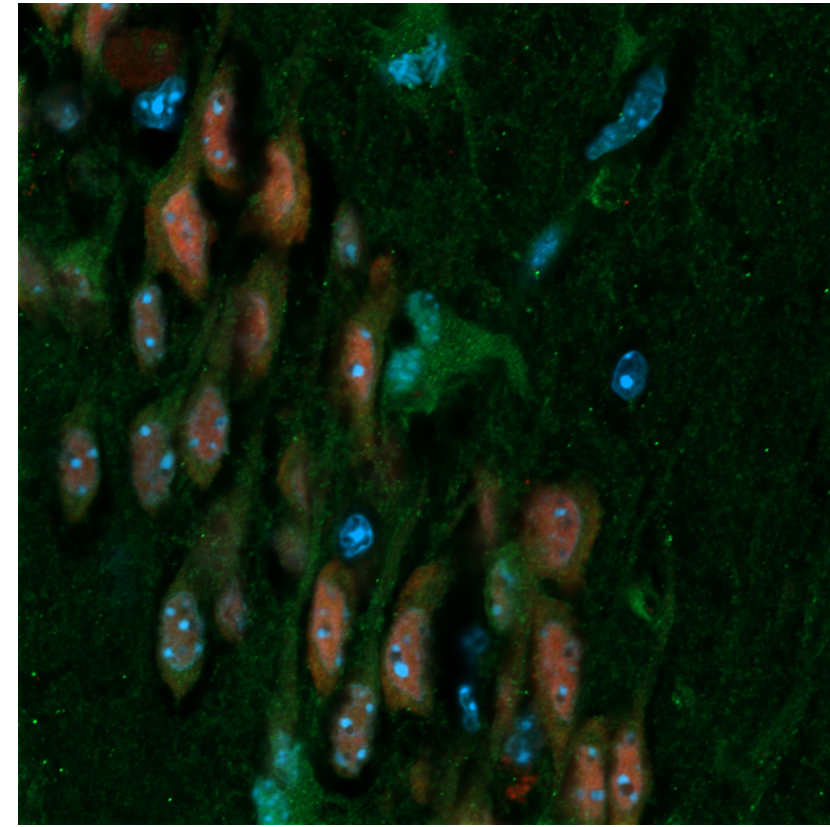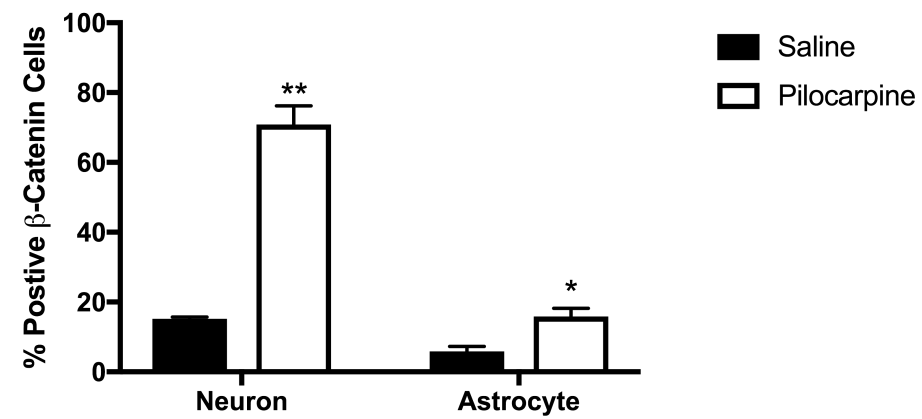

F.

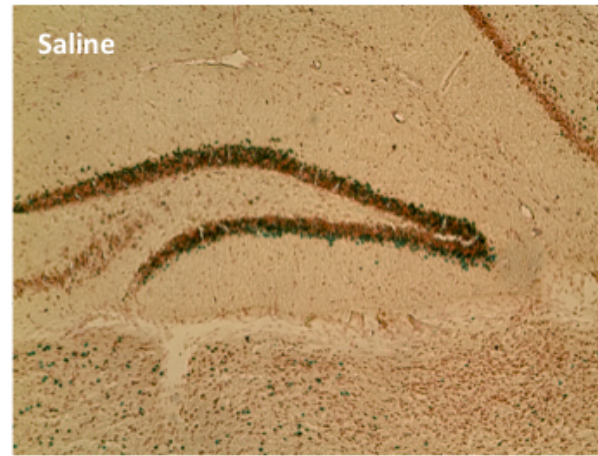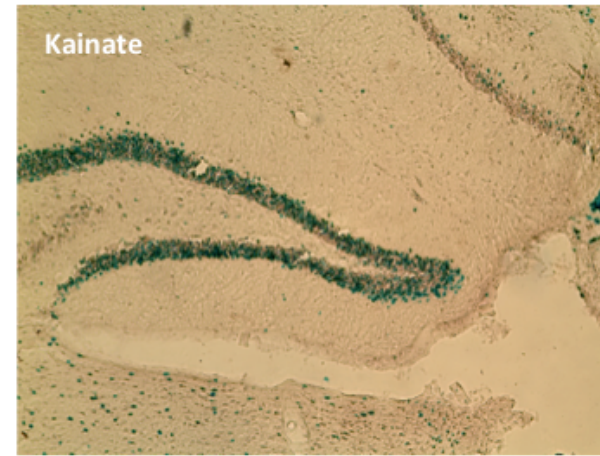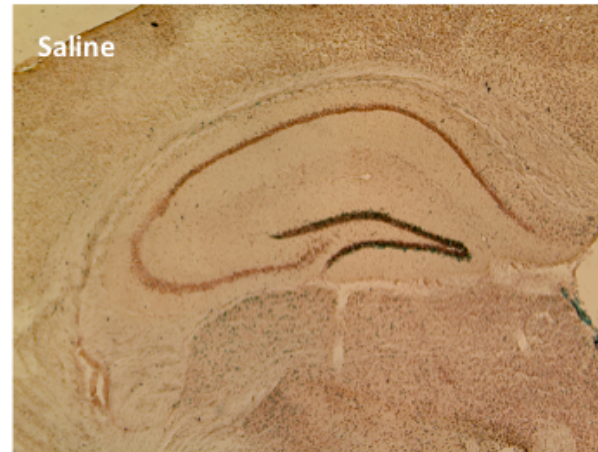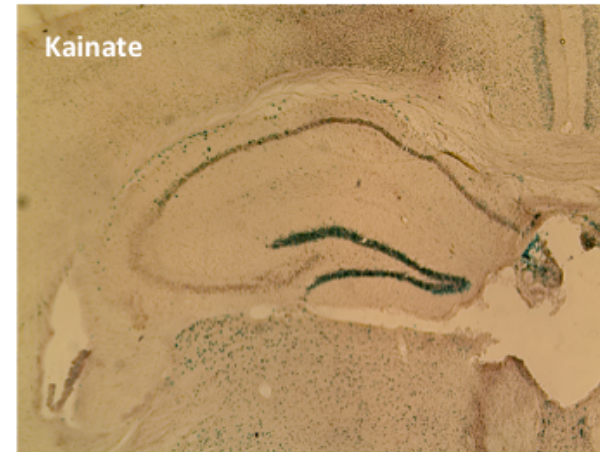

Supplement: S1 Fig — (A) Wnt signaling is activated in the early epileptogenic period using kainate induction in mice. Status epilepticus was induced in male 7-week C57BL6 mice with kainate (30 mg/kg). Representative western blots and quantified data of hippocampal extracts from 3-, 5- or 7-day post-kainate (KA) or control saline (Sal) are shown for pGSK3βS9, GSK3β, β-catenin, and actin (loading control). (n = 4 mice for each group). (B) Wnt signaling is activated in the early epileptogenic period (pilocarpine induction) in rats. Status epilepticus was induced in 4–5 male 7-week Wistar rats (Charles River) with pilocarpine (300 mg/kg). Representative western blots of hippocampal extracts from different time 3-hours, 24-hours, 3-days, or 7-day pilocarpine (Pilo) or control saline (Ctr) are shown for pGSK3βS9, total-GSK3β and tubulin (loading control)-right. Quantified data from the western blots from pilocarpine-treated and saline-treated is shown (left). (C) Expression of Wnt pathway genes. qRT-PCR of Wnt regulators expression in hippocampus following kainate at day 5 post-SE compared to control mice (n = 4–6 mice for each group). Data are represented as mean ± SEM. *P <0.05, **P < 0.01 by unpaired student’s t-test using Prism 9.0 (Graphpad). (D, E) Localization of pGSK3β and of β-catenin in the hippocampus post-SE. pGSK3βS9 (green; D) and β-catenin (green; E) in hippocampus at day 5 post-SE with pilocarpine compared to control mice [Dapi (blue), and NeuN (red)]. Percentage of neuronal and astrocytic positive β-catenin are increased at the DG region of 5-days post-SE with pilocarpine hippocampus when compared to control mice (see supplemental methods). (F) Localization of Wnt Signaling in the hippocampal region following SE induction. We examined the in vivo localization of SE-induced Wnt signaling in mouse hippocampus using the Wnt/β-catenin BAT-GAL reporter mouse [118]. Coronal sections from 5-day control and kainate-SE mice were stained for β-galactosidase activity to determine the l [file pone.0252282.s001.pdf]

A.

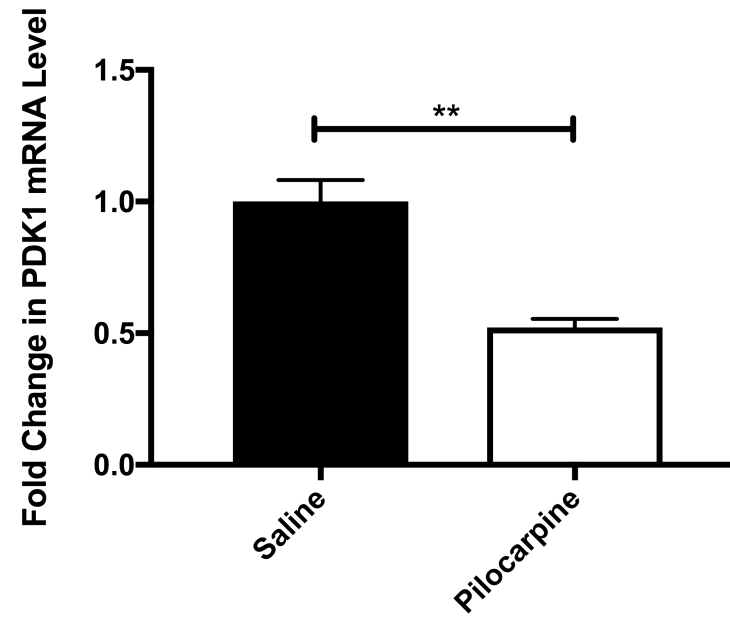

Supplement: S2 Fig — Quantitative RT-PCR of PDK1 expression levels in hippocampus at day 5 following SE with pilocarpine (Pilo) or control (saline). (n = 5–6 mice/group). Data are represented as mean ± SEM. **P <0.01, by unpaired student’s t-test using Prism 9.0 (Graphpad). Refer to supplemental materials for more details on the number of replicates and statistical analyses for each experiment. (PDF) [file pone.0252282.s002.pdf]

A.

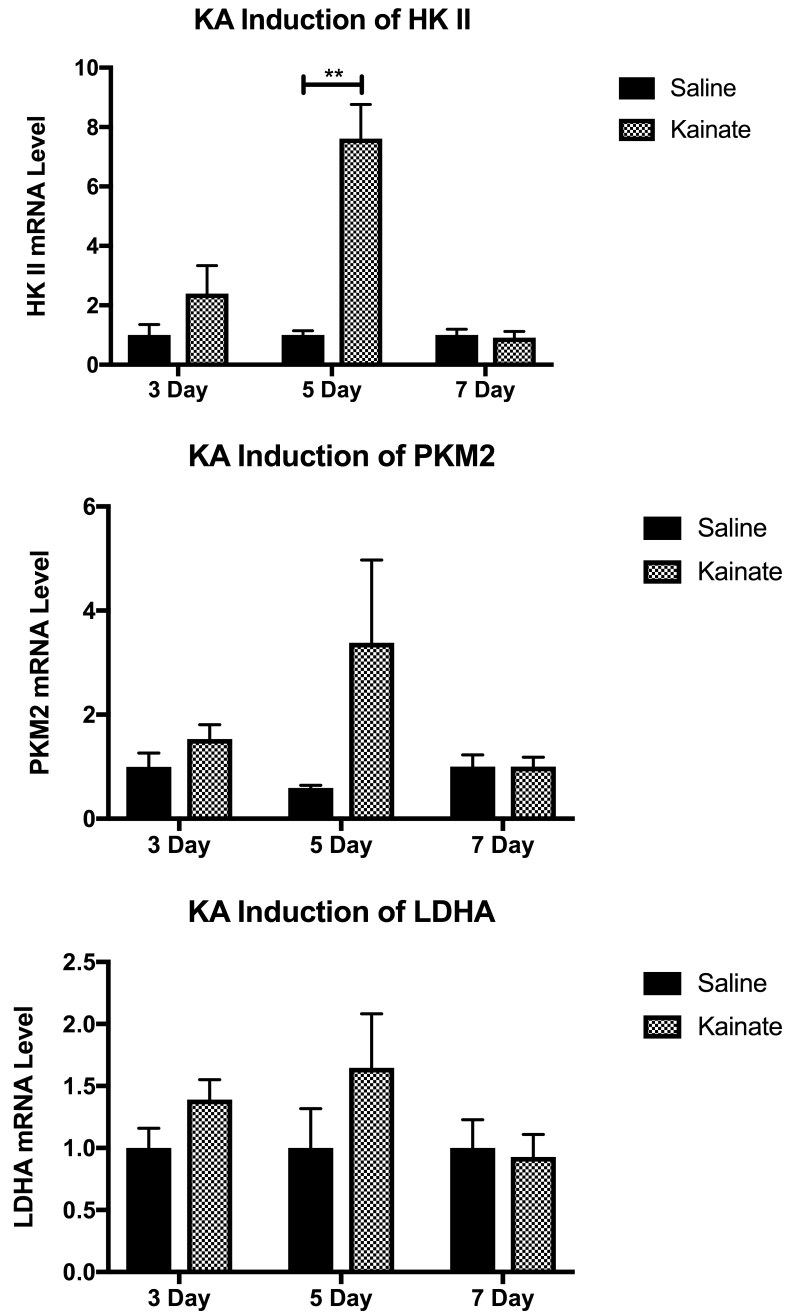

B.

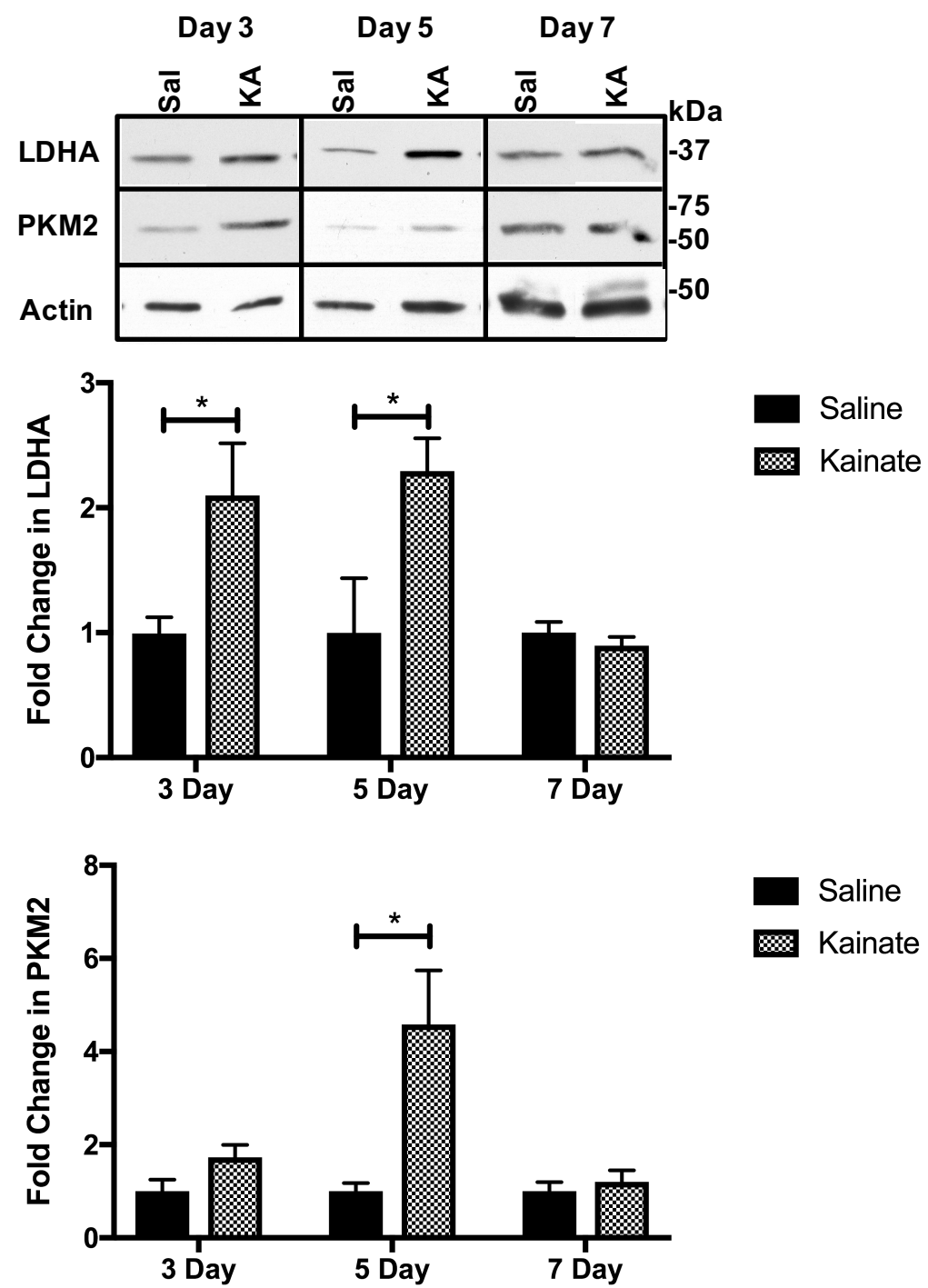

Supplement: S3 Fig — (A) The mRNA expression of Warburg metabolic enzyme isoforms is elevated after kainate-induced SE. Quantitative RT-PCR detection of HK II, PKM2 and LDHA gene expression levels from the hippocampi of the 3-, 5- and 7-day post-status epilepticus mice in (kainate treated) compared to control (saline) mice. (B) The protein expression of Warburg metabolic enzyme isoforms is elevated after kainate-induced SE. Representative and quantified western blots of hippocampal extracts from 3-, 5- or 7-day kainate (KA), or control saline (Sal) are shown for PKM2, LDHA, and actin (loading control). (n = 3–5 for each group). Data are represented as mean ± SEM. *P <0.05, **P < 0.01 by unpaired student’s t-test using Prism 9.0 (Graphpad). Refer to supplemental materials for more details on the number of replicates and statistical analyses for each experiment. (PDF) [file pone.0252282.s003.pdf]

A.

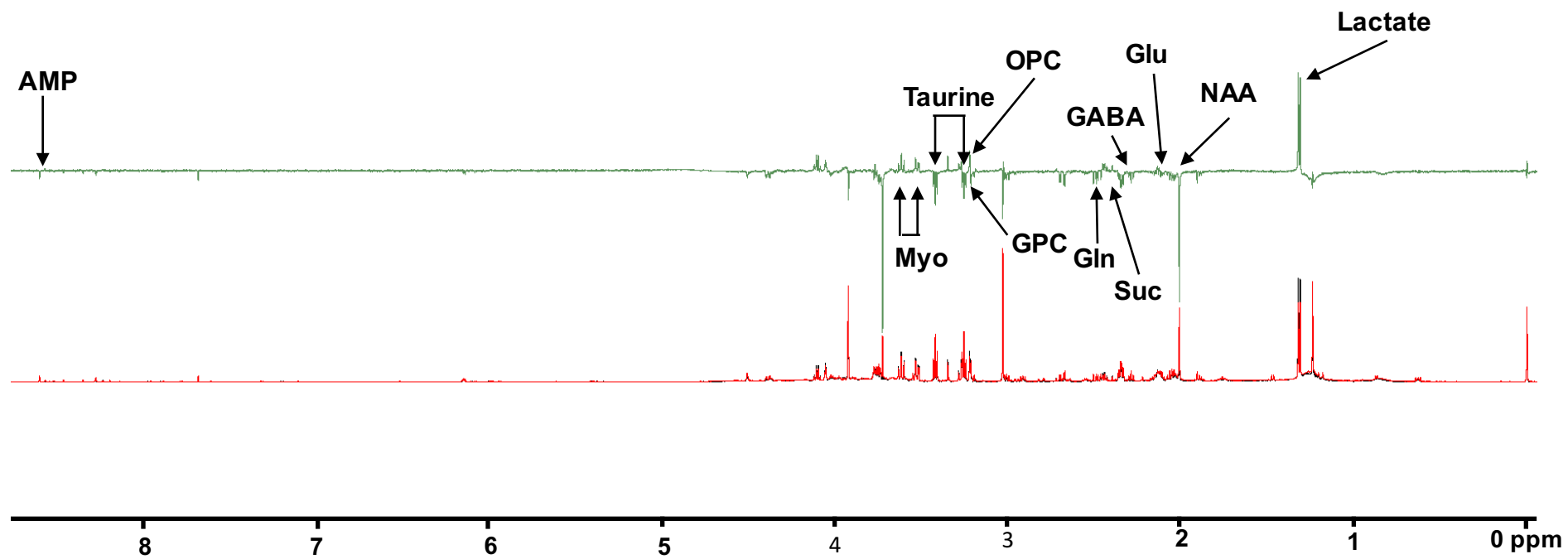

B.

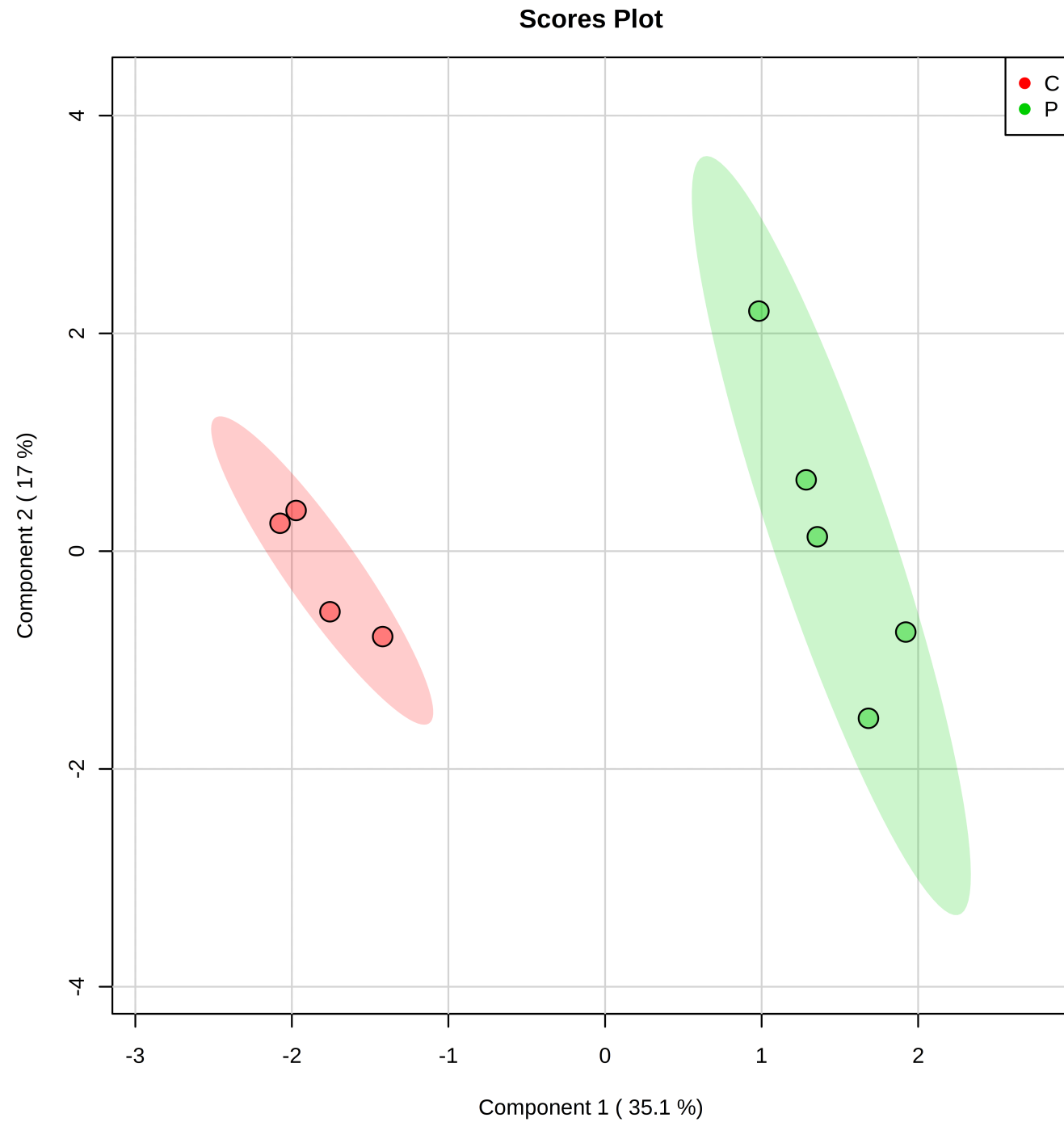

C.

### Top 25 compounds correlated with the Lactate

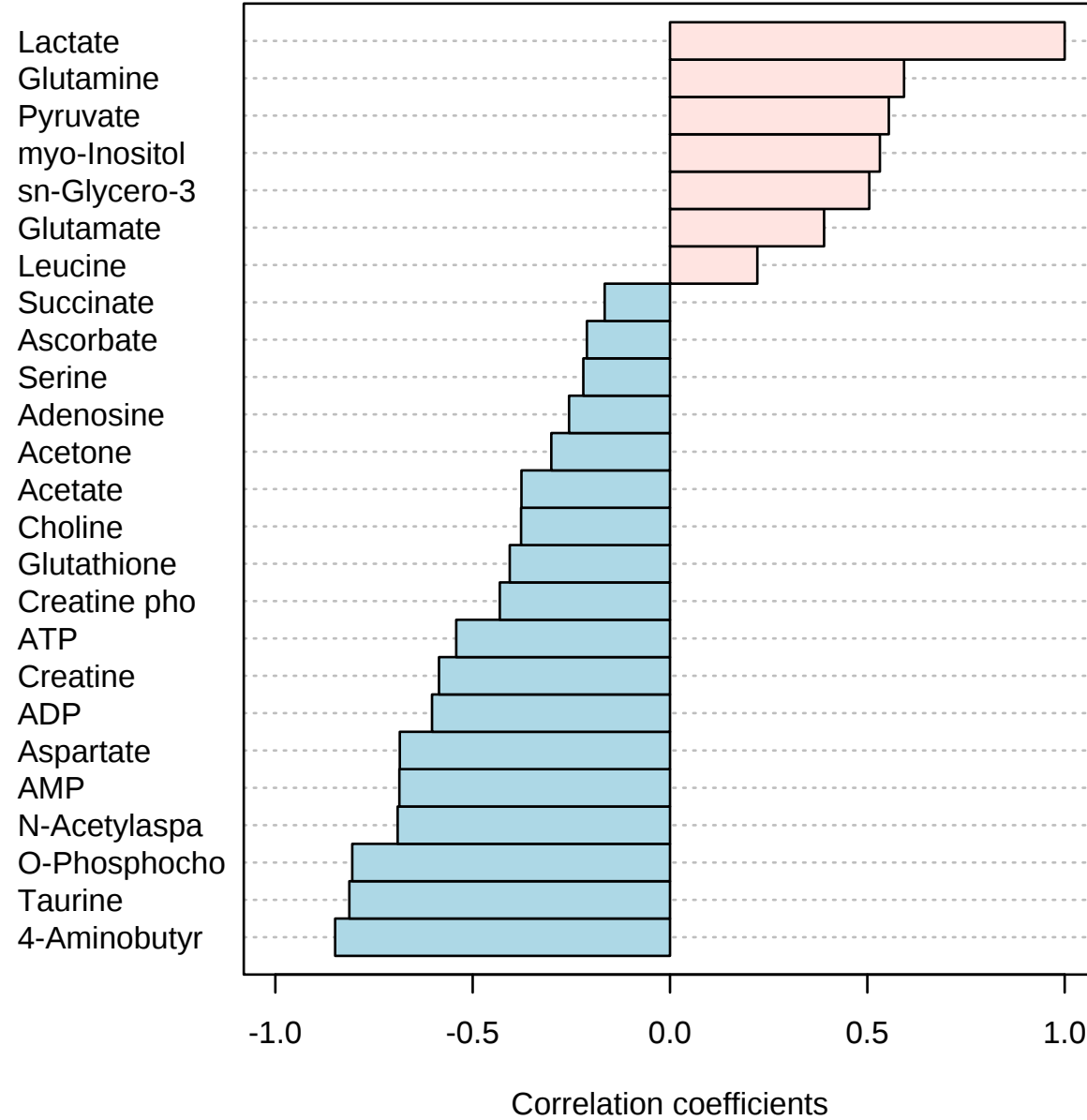

D.

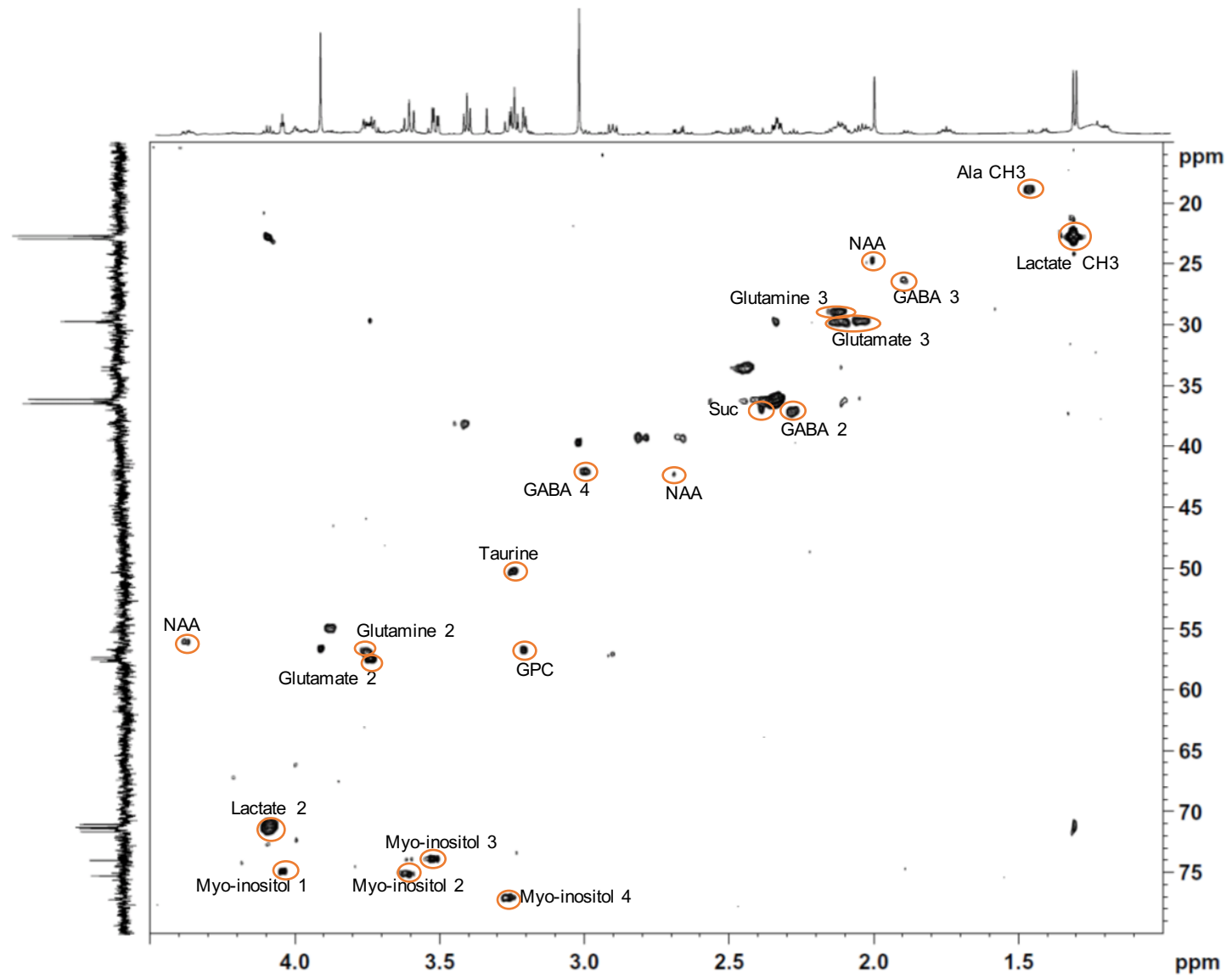

E.

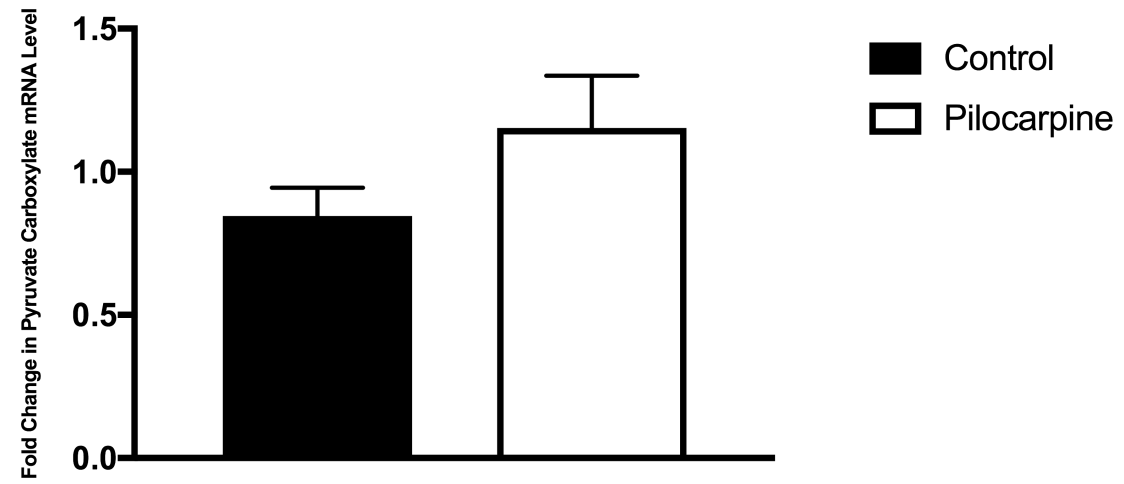

Supplement: S4 Fig — (A) Representative 1H NMR spectra of hippocampal metabolites. Red: Control, Black: Pilocarpine, Green: Difference spectrum (scaled 4x). The NMR peaks corresponding to significantly changed levels of metabolites are labeled. A non-significant resonance at 3.716 ppm was removed for clarity. Non-standard abbreviations used are Suc, succinate; GPC, sn-glycero-3-phosphocholine; Myo, myo-inositol; OPC, O-phosphocholine; GABA, 4-aminobutyrate; NAA, N-acetylaspartate. (B) Partial least squares analysis demonstrates significant difference between control and 5-day post-SE metabolism. The metabolites showed significant separation (>95% confidence) between control mice (red) and pilocarpine-treated mice (green). (C) Metabolite changes correlated and anti-correlated with lactate in control and 5-day post-SE metabolism. The top 25 metabolites pro- and anti-correlated with lactate are shown along with their correlation coefficients calculated in Metaboanalyst 4.0 [62, 63]. (D) Representative 1H-13C HSQC NMR spectrum used for identification of hippocampal metabolites after infusion of [U-13C]-glucose and administration of pilocarpine. The NMR peaks corresponding to significantly changed levels of metabolites are circled and labeled. Abbreviations used: Suc, succinate; GPC, sn-glycero-3-phosphocholine; Myo, myo-inositol; GABA, 4-aminobutyrate; NAA, N-acetylaspartate. (E) Quantitative RT-PCR of Pyruvate Carboxylase (PC) expression levels in hippocampus at day 5 following SE with pilocarpine (Pilo) or control (saline). (n = 4–6 mice/group). Data are represented as mean ± SEM. P >0.05, by unpaired student’s t-test using Prism 9.0 (Graphpad). Refer to supplemental materials for more details on the number of replicates and statistical analyses for each experiment. (PDF) [file pone.0252282.s004.pdf]

A.

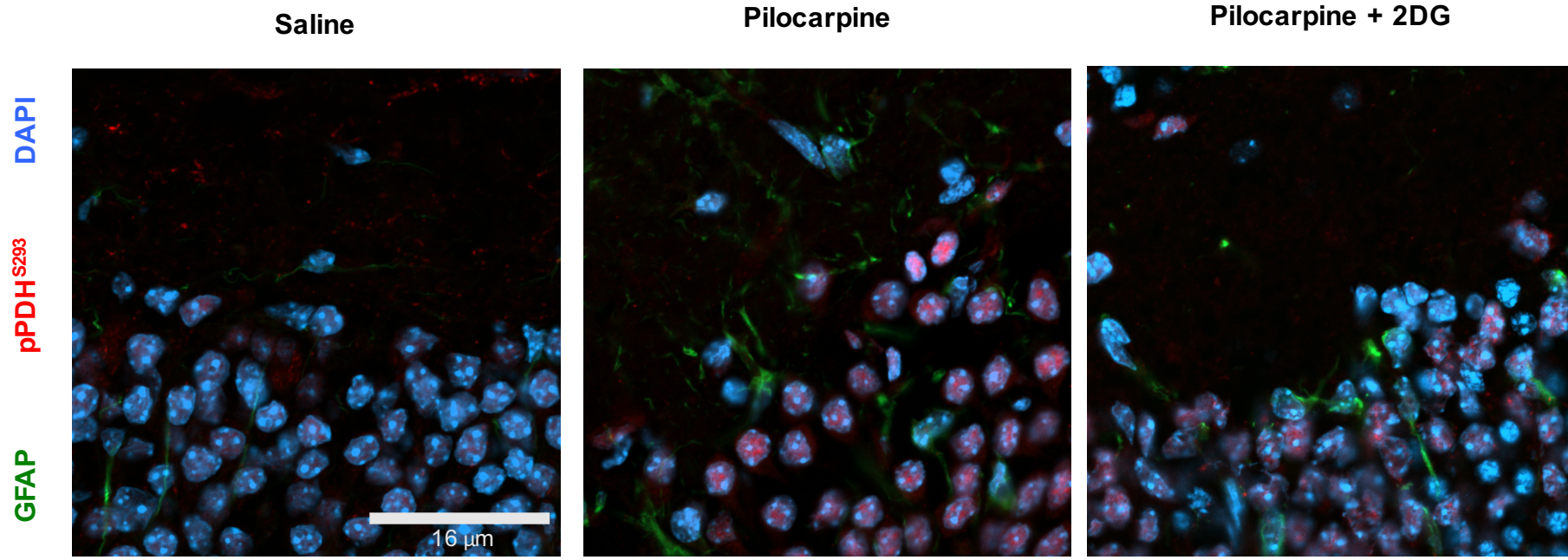

Supplement: S5 Fig — (A) Confocal images (X63) of positive pPDHS293 (red) neuronal cells that are not changed in the DG region of 5 days post-SE in hippocampus 2-DG-treated pilocarpine mice when compared to control pilocarpine mice. [GFAP (green) and Dapi (blue)]. (PDF) [file pone.0252282.s005.pdf]

A.

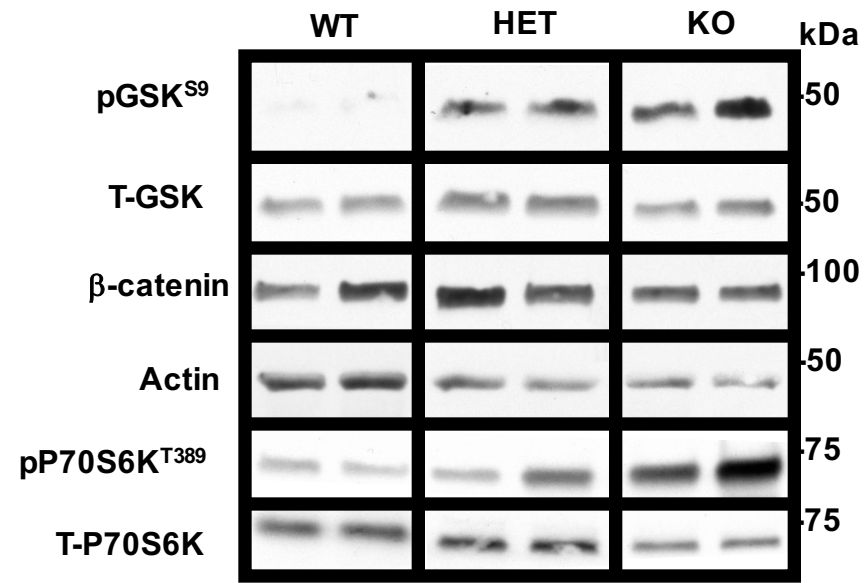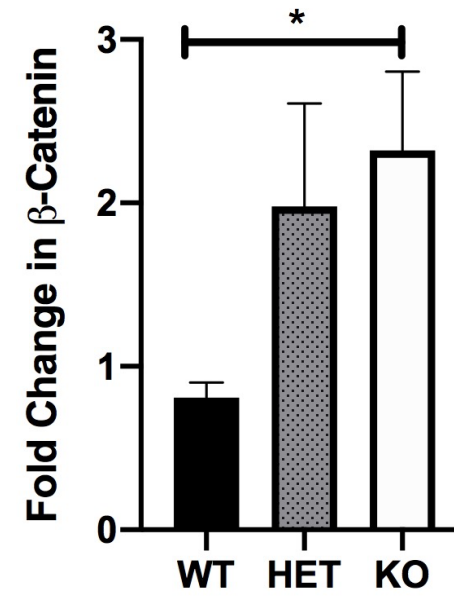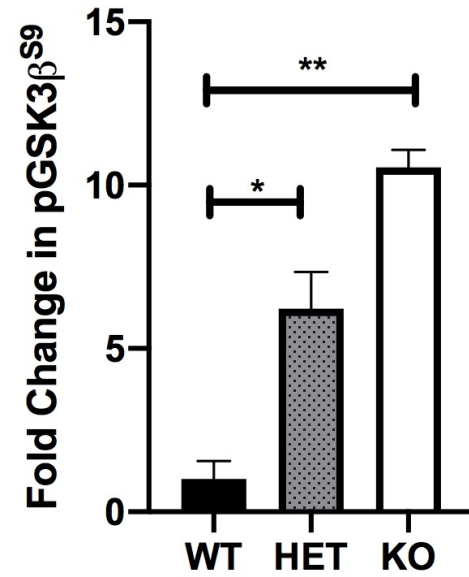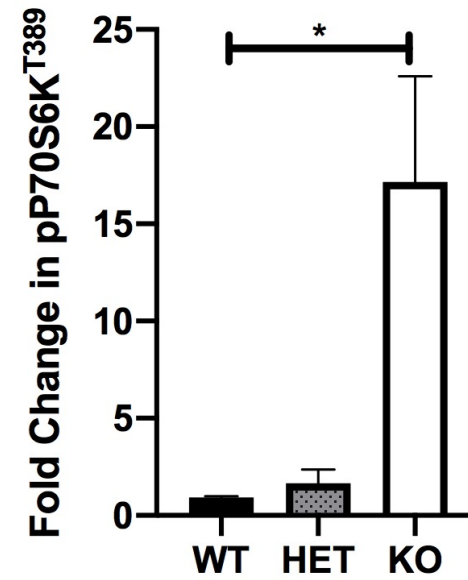

B.

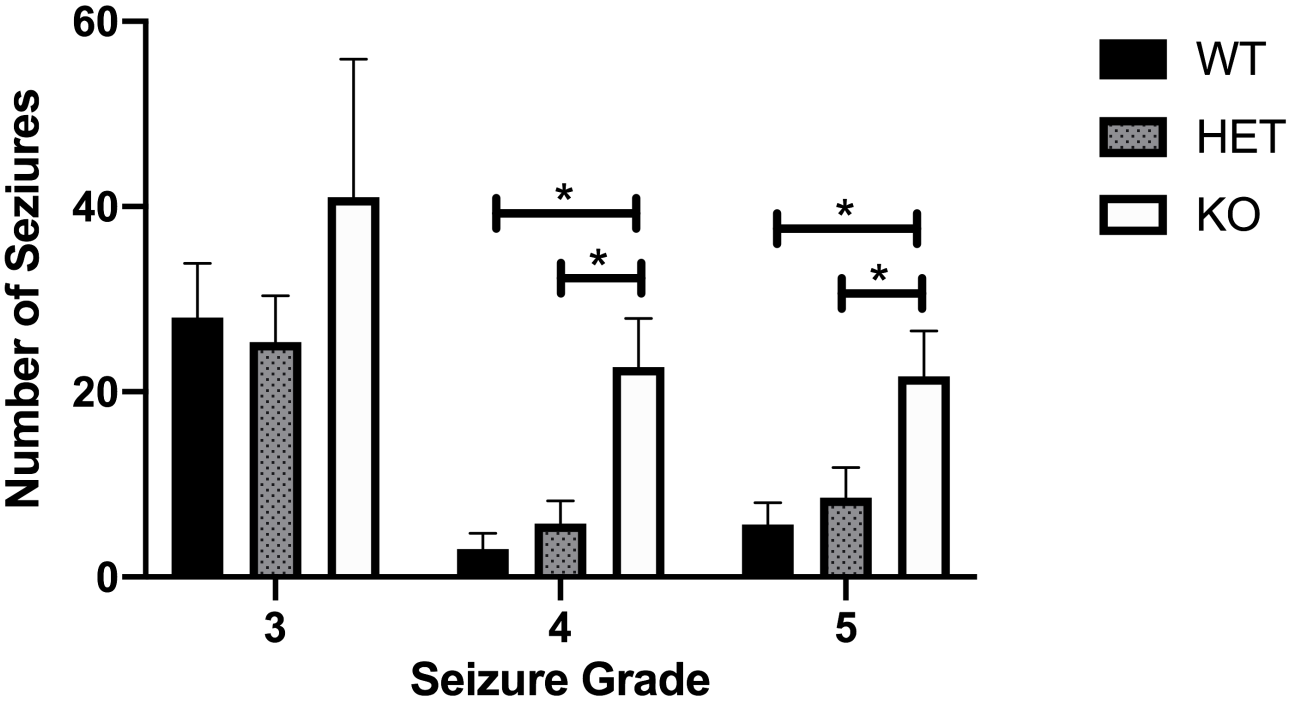

Supplement: S6 Fig — (A) Representative western blot with quantified data of baseline levels of Wnt and mTOR signaling markers in C57BL/6 mouse strain were increased in HBP1-/- Knockout (KO) and HBP1+/- heterozygous (HET) when compared to control (WT). (B) HBP1-/- KO mice have increased number and severity of seizures after seizure induction based on the Racine scale [73] (n = 3–5 mice for each group). Data are represented as mean ± SEM. *P <0.05 by unpaired student’s t-test using Prism 9.0 (Graphpad). Refer to supplemental materials for more details on the number of replicates and statistical analyses for this experiment. (PDF) [file pone.0252282.s006.pdf]
