# Supplementary material for: A Warburg-like metabolic program coordinates Wnt, AMPK, and mTOR signaling pathways in epileptogenesis
Source: PLoS One. 2021 Aug 6;16(8):e0252282. doi: 10.1371/journal.pone.0252282 (PMC8345866; doi:10.1371/journal.pone.0252282)

Raw Data of Western Blots

Raw data of figures are shown in order. All blots were captured with Chemiluminescent high sensitivity mode that quantitated by the Bio-Rad Chemidoc MP Imaging System

Fig. 1A
Fig. 2A
Fig. 2C
Fig. 3B
Fig. 5A
Fig. 5E
Fig. 6A
Fig. 6B
Fig. 6C
Fig. 7B
Fig. 7F
Fig. 7H
S. Fig. 1A
S. Fig. 3B
S. Fig. 6A

Figure 1 A Blots

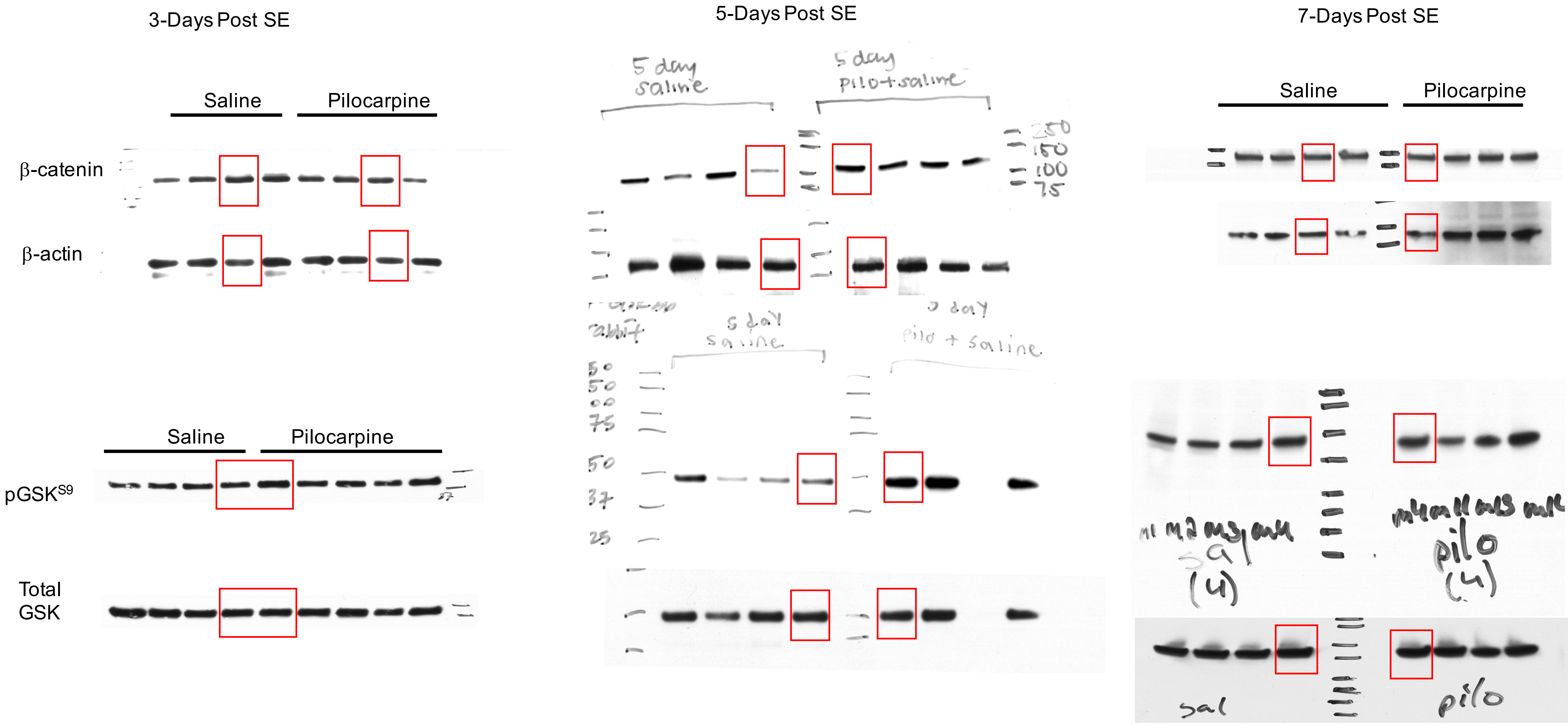

Figure 2 A Blots

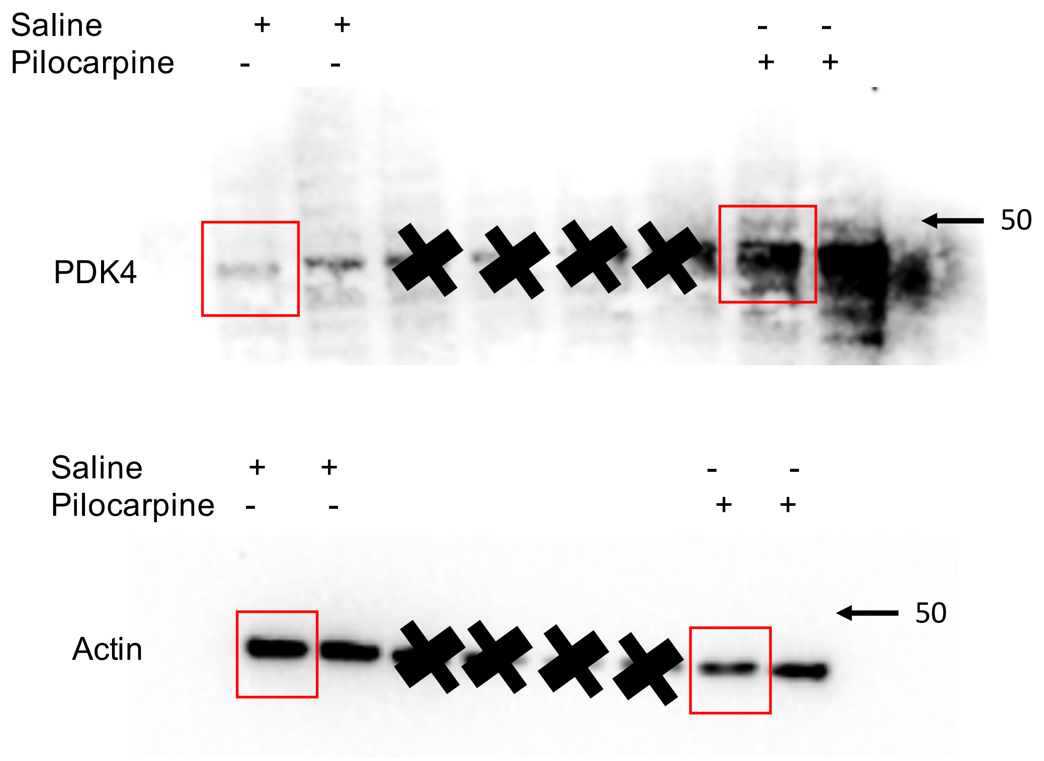

Figure 2 C Blots

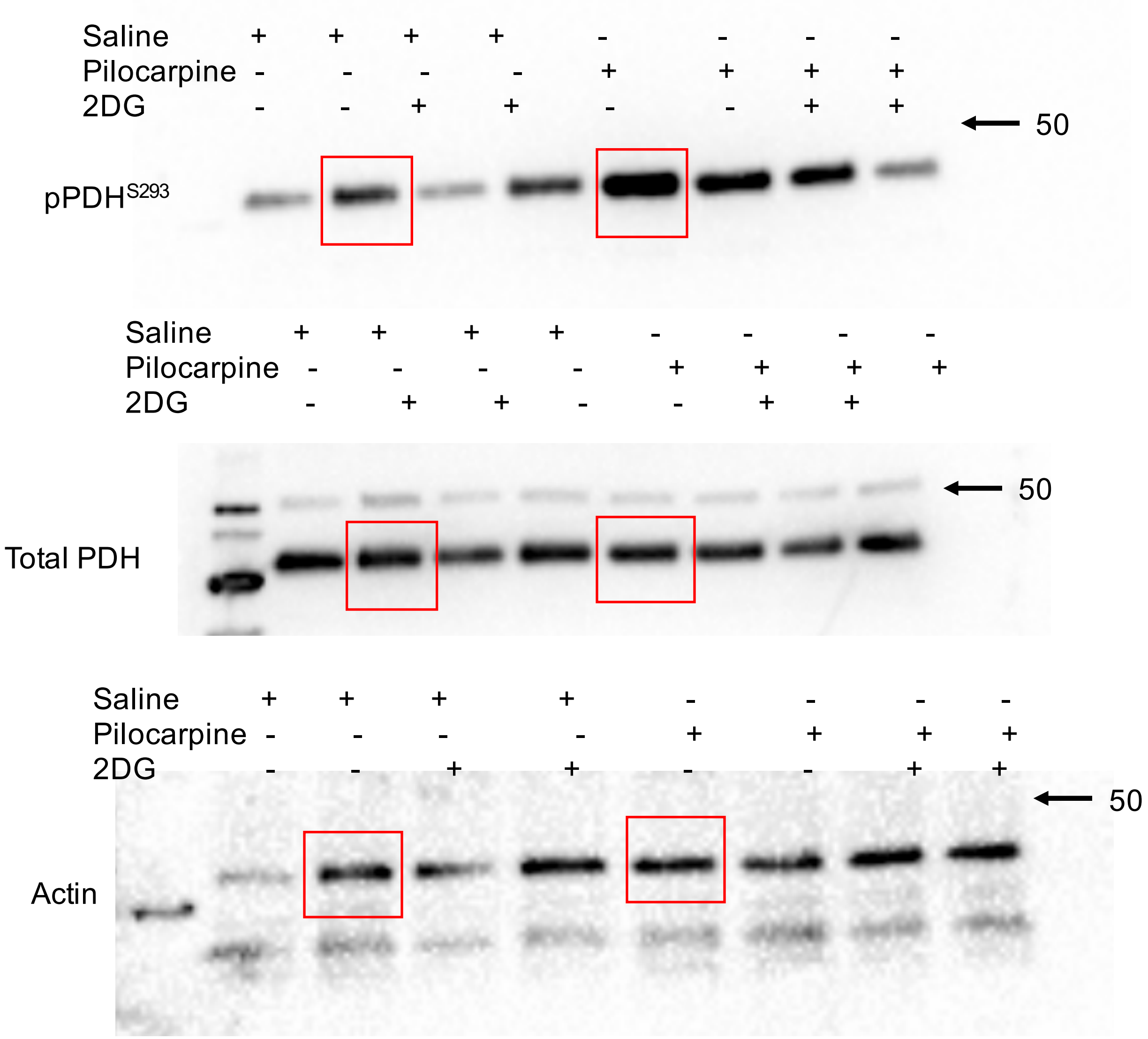

Figure 3 B Blots

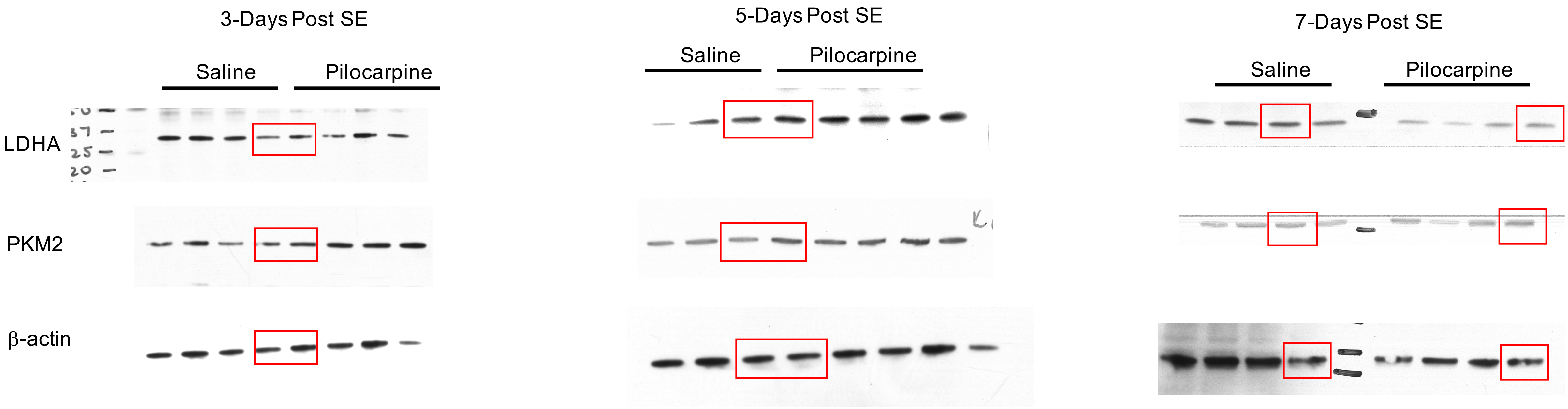

Figure 5 A Blots

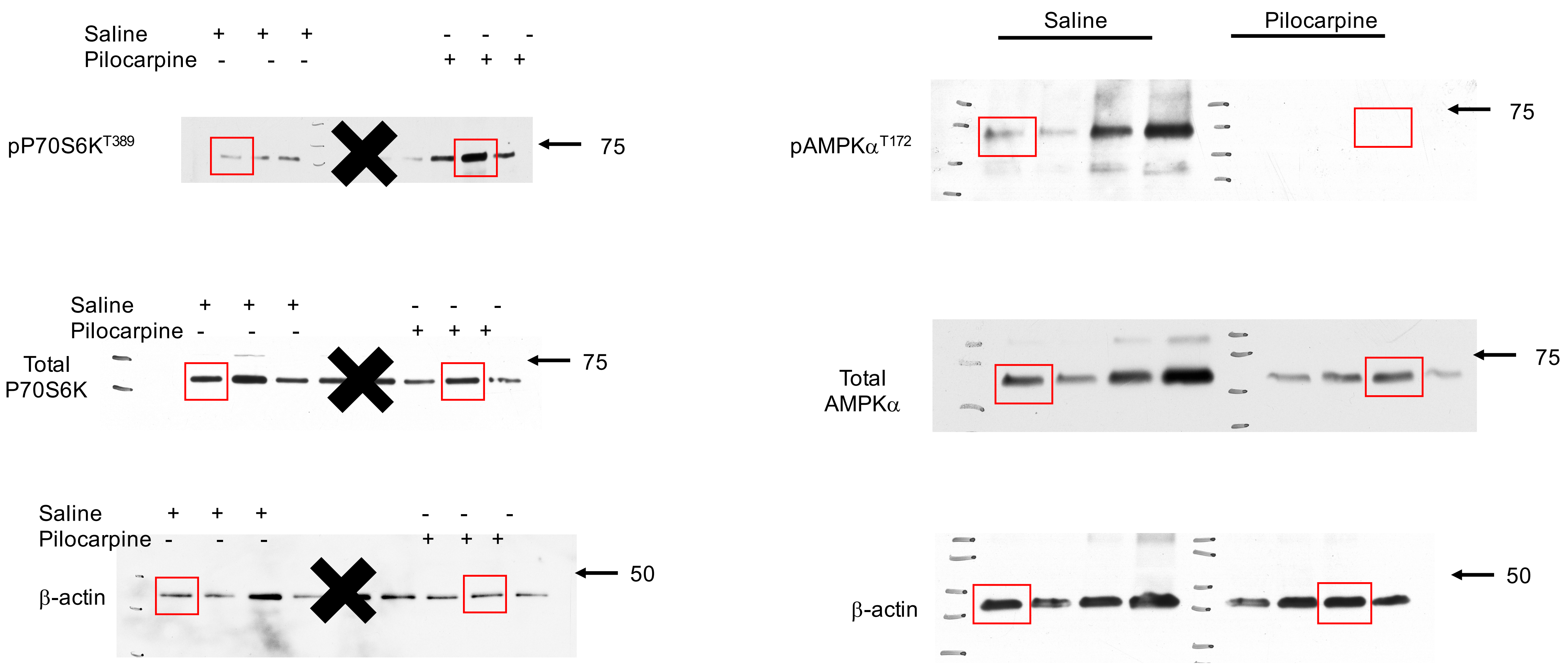

Figure 5 E Blots

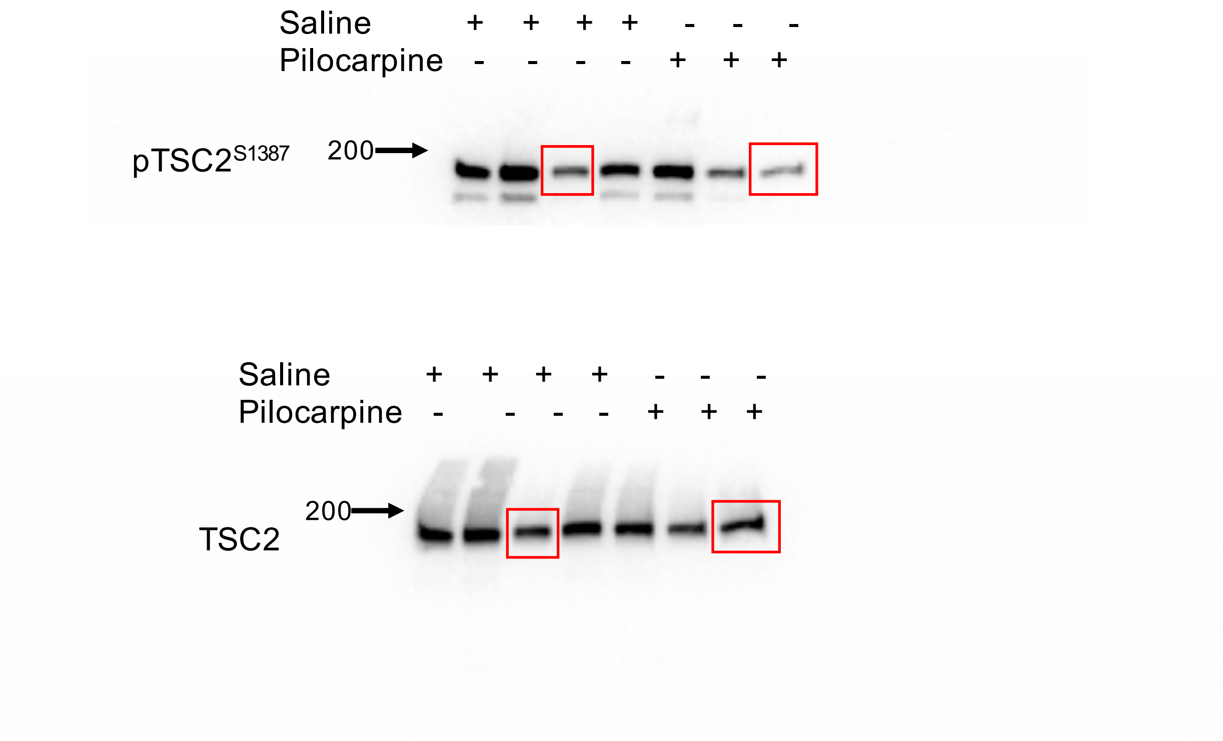

Figure 6 A Blots

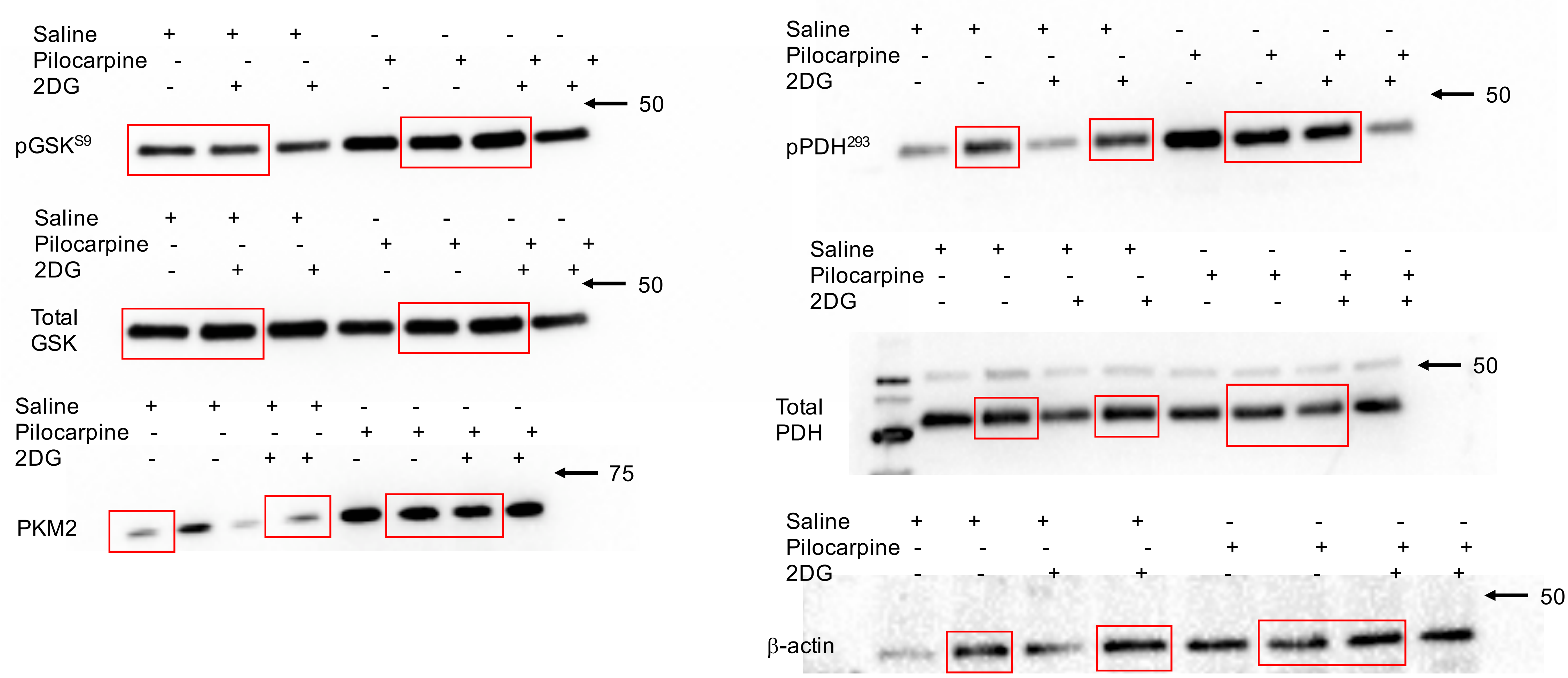

Figure 6 B Blots

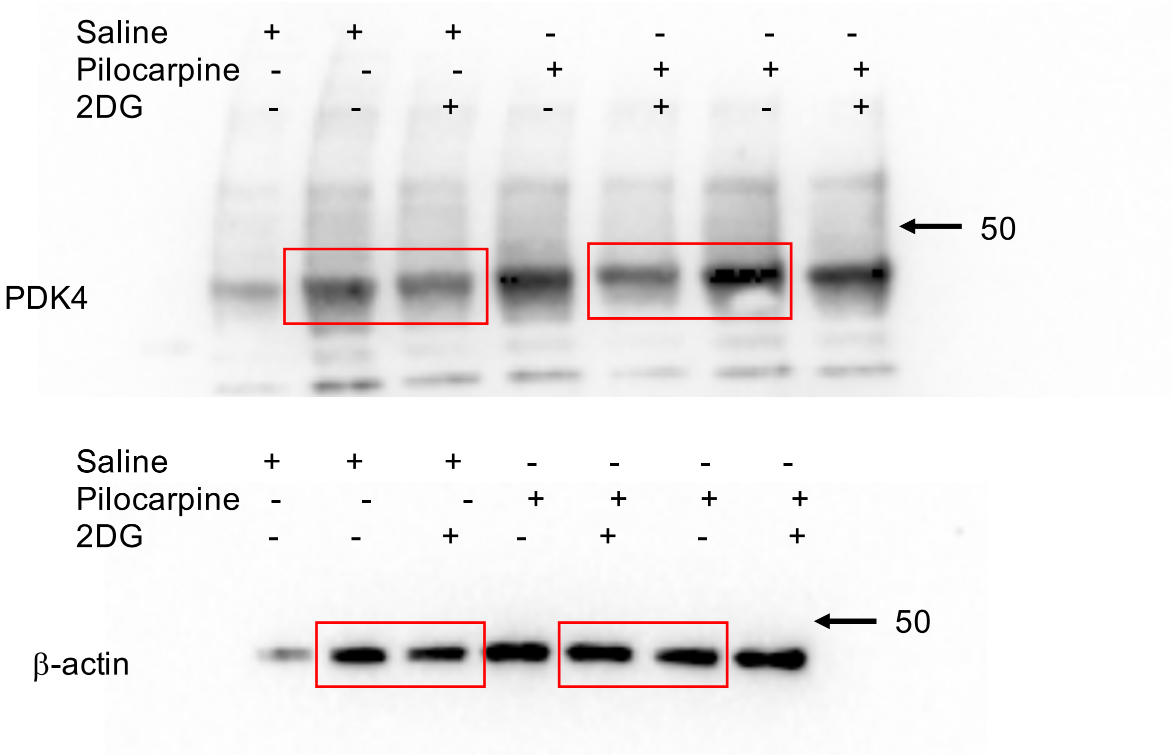

Figure 6 C Blots

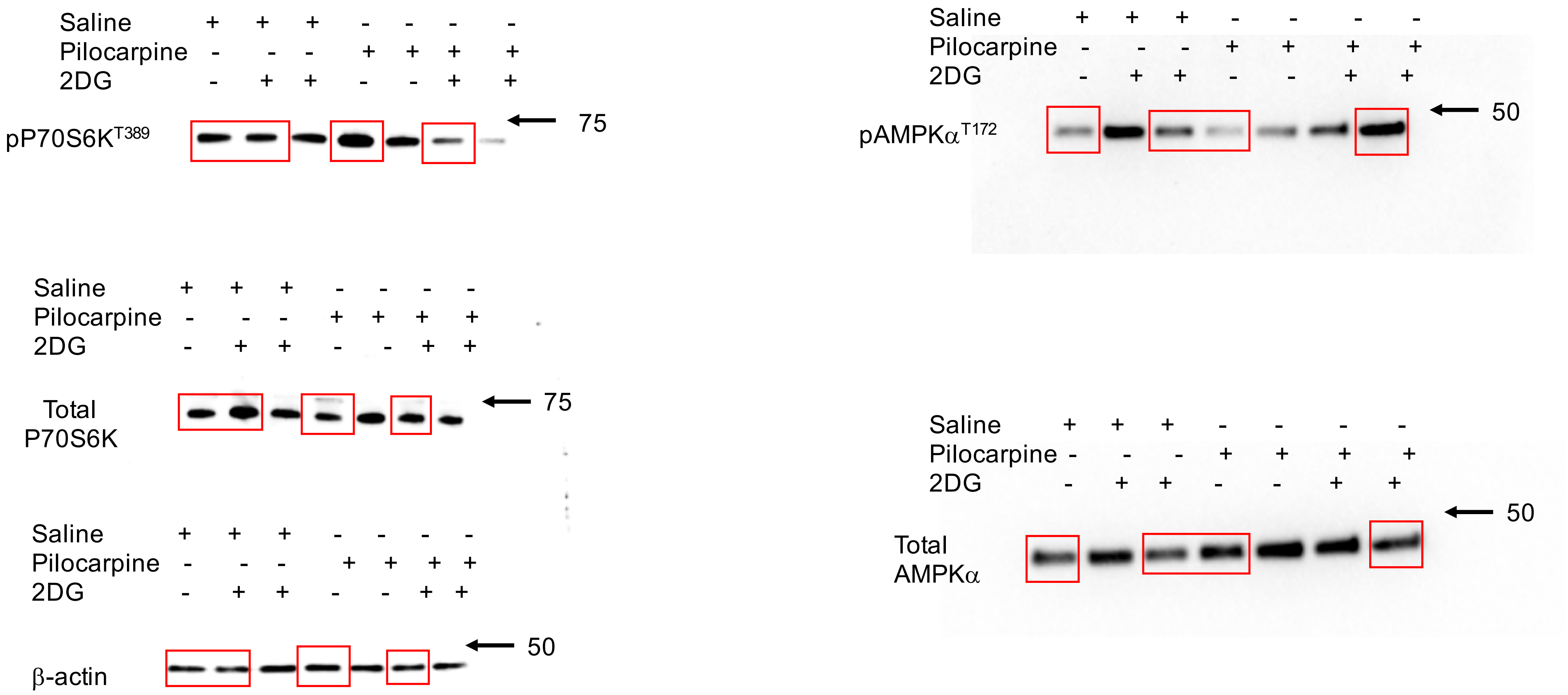

Figure 7 B Blots

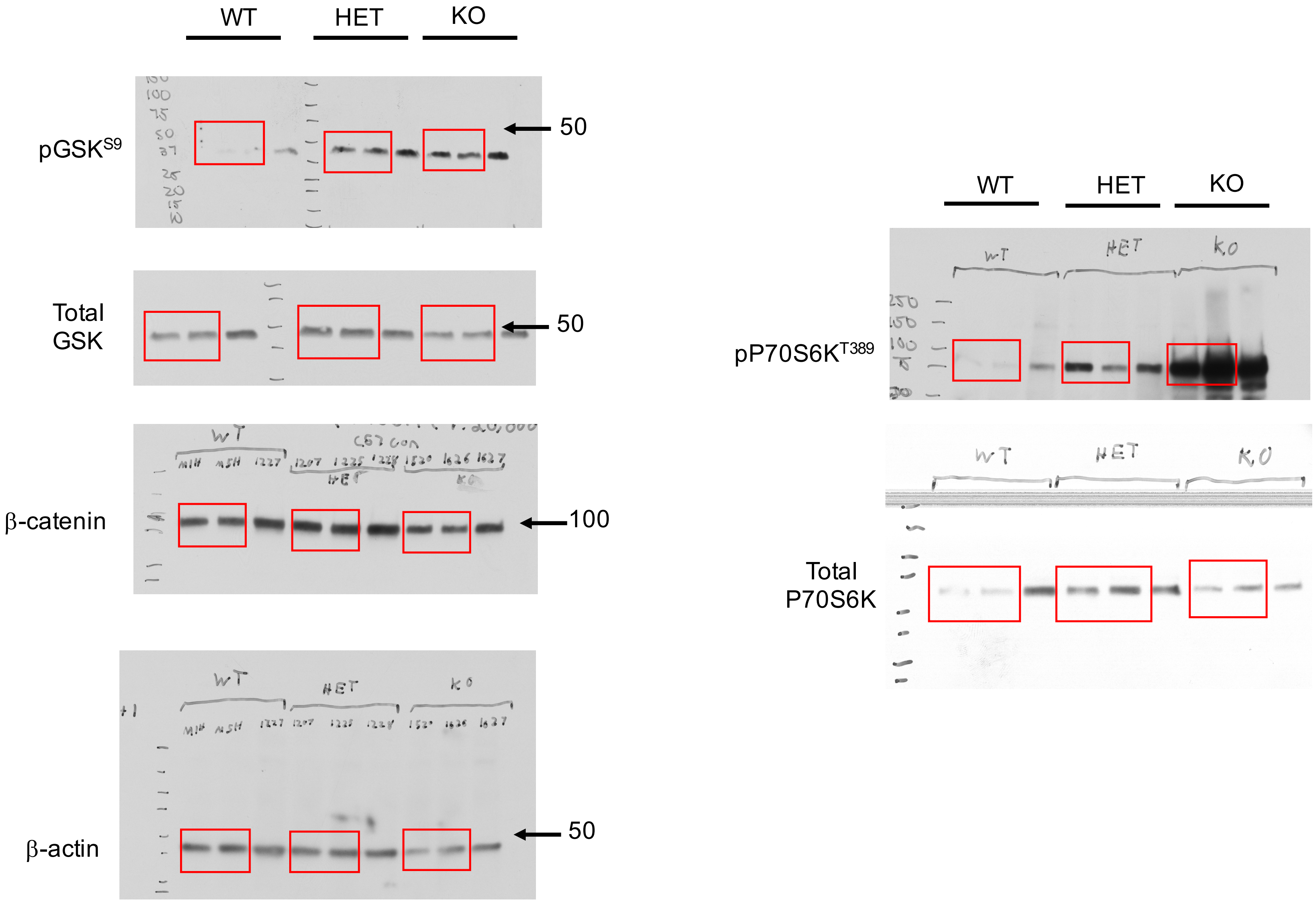

Figure 7 F Blots

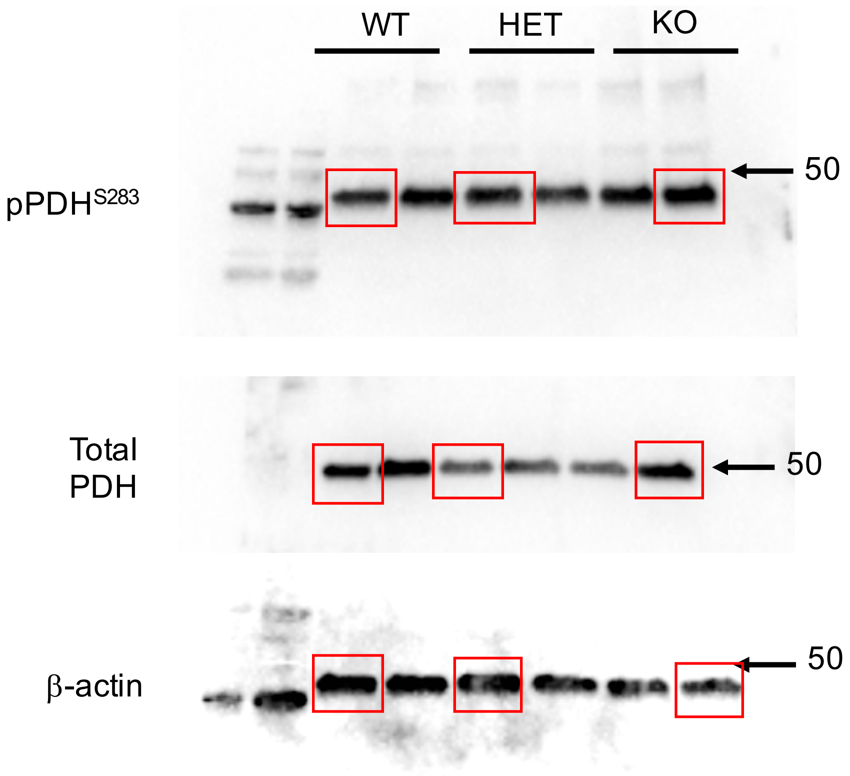

Figure 7 H Blots

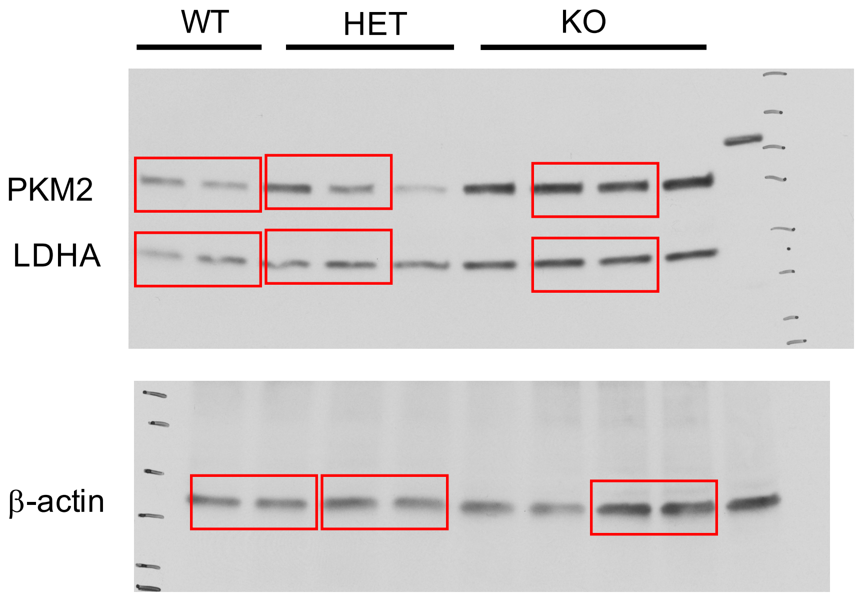

Supp. Figure 1 A Blots

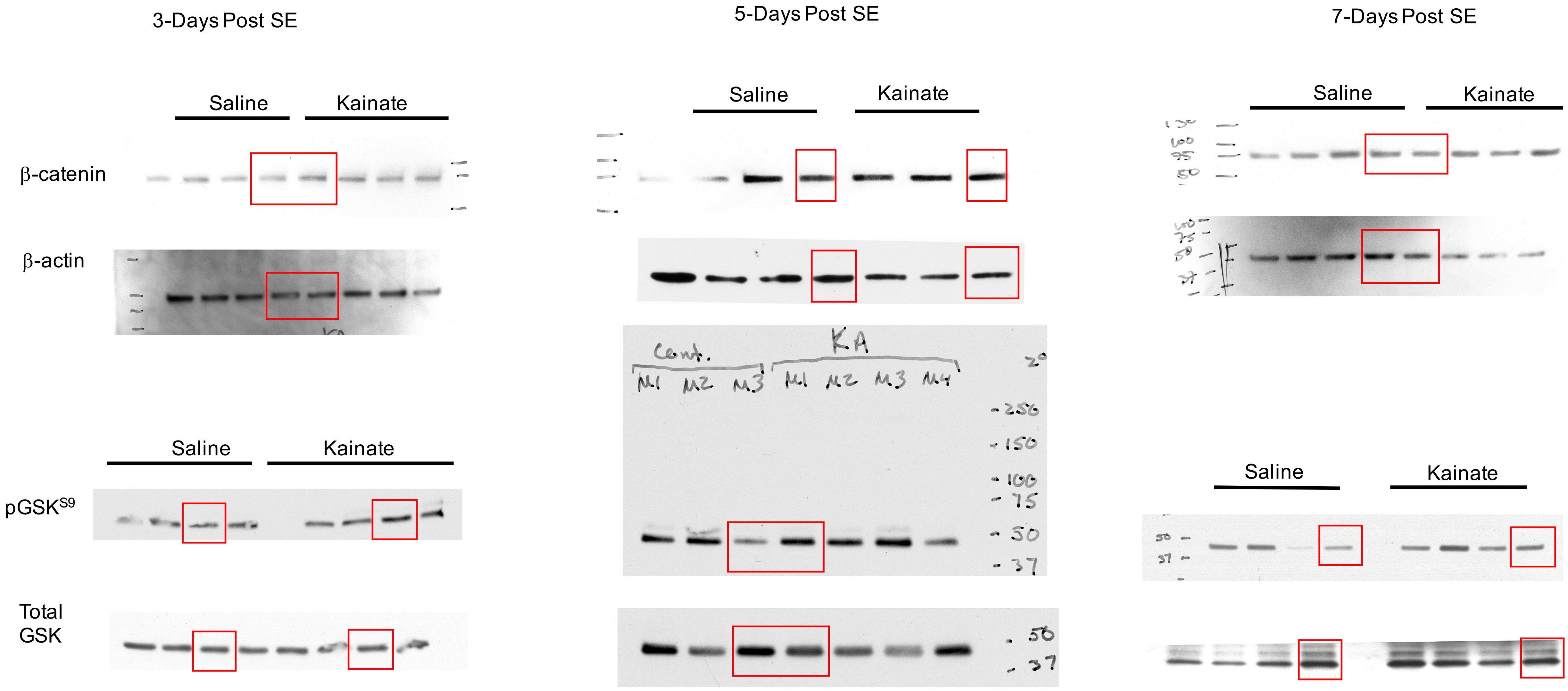

Supp. Figure 3 B Blots

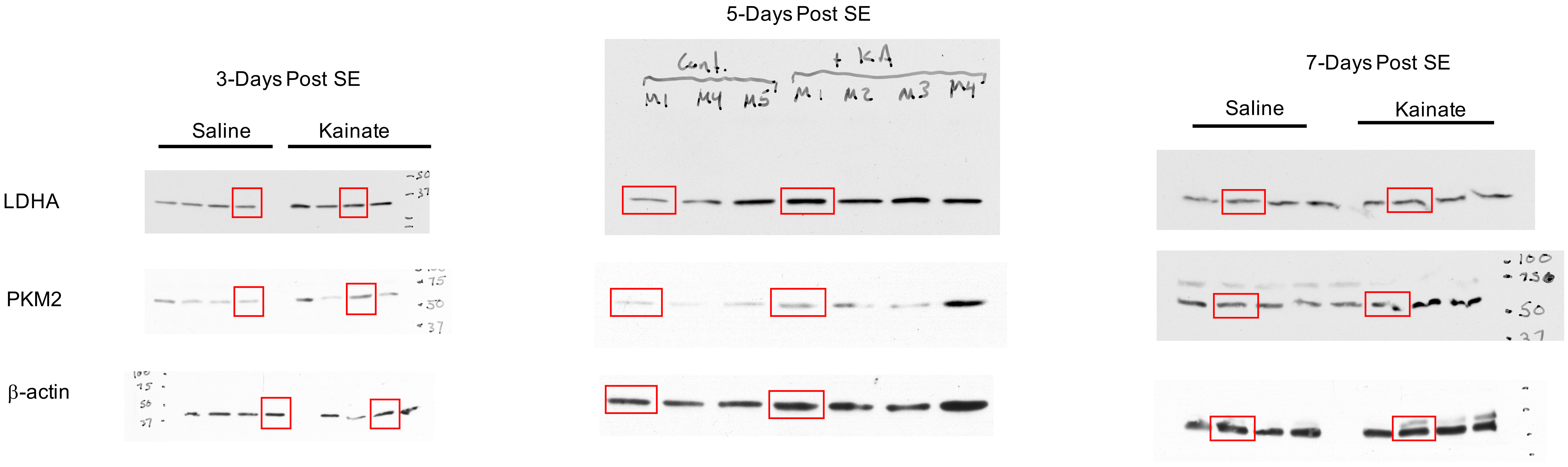

Supp. Figure 6 A Blots

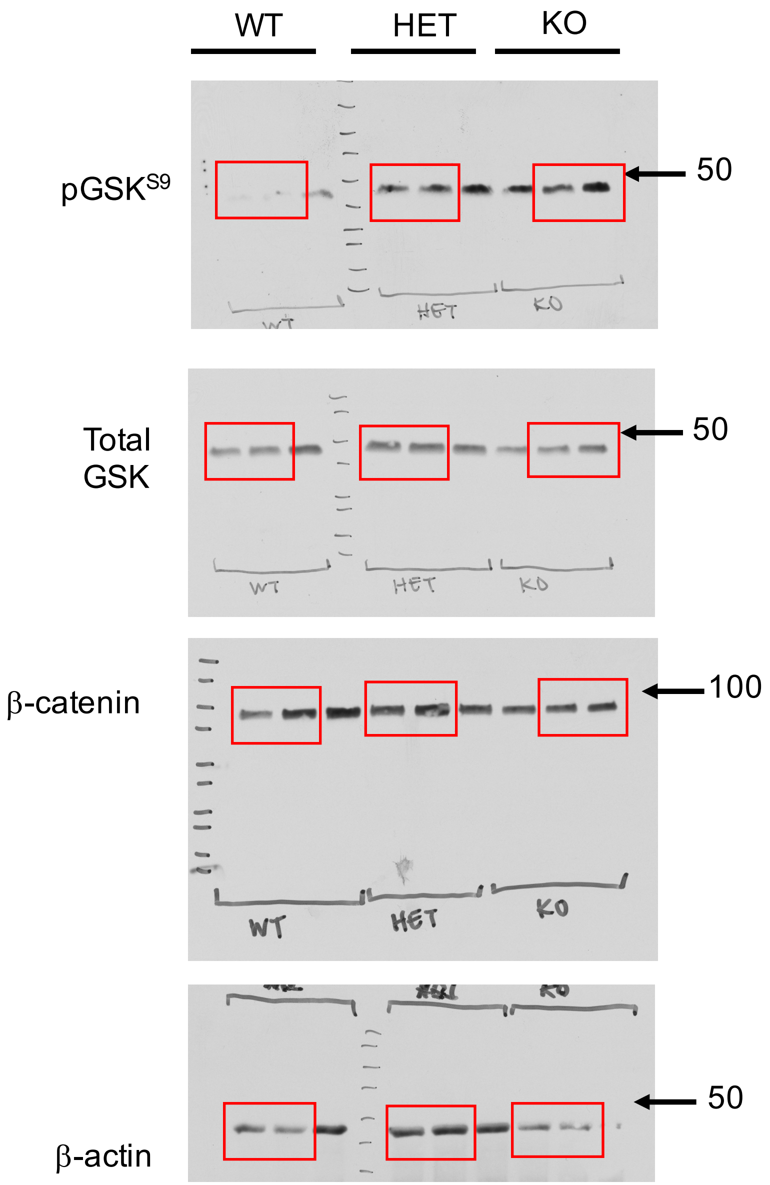

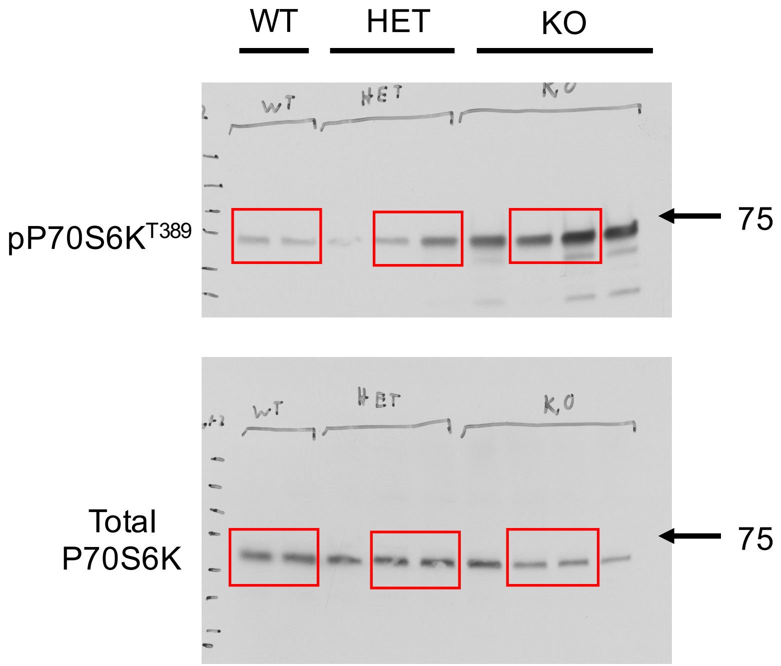

Supplement: S1 File — (PDF) [file pone.0252282.s009.pdf]
