# Supplementary material for: A Warburg-like metabolic program coordinates Wnt, AMPK, and mTOR signaling pathways in epileptogenesis
Source: PLoS One. 2021 Aug 6;16(8):e0252282. doi: 10.1371/journal.pone.0252282 (PMC8345866; doi:10.1371/journal.pone.0252282)
Supplement: S2 File — (DOCX) [file pone.0252282.s010.docx]

# Supplemental Materials:

## Oligonucleotides:

| **Name** | **Forward** | **Reverse** |
| --- | --- | --- |
| **Axin-2** | GAC AGT AGC GTA GAT GGA GTC C | CGG CTT TCC AGC TCC AGT TT |
| **FOXG1** | GCT TTG AGC CAG TGA GGA TT | ATG GTT TGG TGG GGA GGT TG |
| **GAD67** | TAG CCT GGA AGA GAA GAG TCG | GTT TGC TCC TCC CCG TTC TT |
| **GLS** | ATG GAG CAG CGG GAT TAT GAC | ATG GTG TCC AAA GTG TAG TGC TT |
| **GS** | CAG GGT GAG AAA GTC CAA GC | CTC GAA ACA TGG CAA CAG GA |
| **HBP1** | GGA AGA CTT TGC TAG AGC CG | CAG TGA GCA AGC CAT CTT CT |
| **HK2** | TCG GTT TCT CTA TTT GGC CCC | GGT AGC TCC TAG CCC CTT CT |
| **LDHA** | AAT GAA GGA CTT GGC GGA TG | GCT TGG AGT TCG CAG TTA CAC |
| **PC** | CAG GGC GGA GCT AAC ATC TAC | TAT ACT CCA GAC GCC GGA CA |
| **PDK1** | TTC TGC GAC AAG AGT TGC CT | TGT GCC GGT TTC TGA TCC TT |
| **PDK4** | AAA GAT GCT CTG CGA CCA GT | GGG TCA AGG AAG GAC GGT TT |
| **PKM2** | CCA CTT GCA GCT ATT CGA GG | GTC ACG GCA ATG ATA GGA GC |
| **SFRP1** | ACT GGC CCG AGA TGC TCA AA | CAT CCT CAG TGC AAA CTC GCT |
| **Wnt8b** | CCG TGT GCG TTC TTC TAG TCA | TTC CAA CGG TCC CAA GCA AA |

## Antibody Reagents:

| **Reagent** | **Source** | **Identifier** |
| --- | --- | --- |
| β**-actin** | Sigma-Aldrich | Cat. # A2228 |
| **Alexa 488 Donkey Anti-Goat** | Jackson Immunoresearch | Cat. # 705-546-147 |
| **Alexa 488 Donkey Anti-Mouse** | Jackson Immunoresearch | Cat. # 715-586-150 |
| **Alexa 488 Donkey Anti-Rabbit** | Jackson Immunoresearch | Cat. # 711-546-152 |
| **Alexa 594 Donkey Anti-Goat** | Jackson Immunoresearch | Cat. # 705-586-147 |
| **Alexa 594 Donkey Anti-Mouse** | Jackson Immunoresearch | Cat. # 715-546-150 |
| **Alexa 594 Donkey Anti-Rabbit** | Jackson Immunoresearch | Cat. # 711-586-152 |
| β**-catenin** | Millipore | Cat. # 06-734 |
| **DAPI** | Abcam | Cat. # ab104139 |
| **GFAP** | Abcam | Cat. # ab53554 |
| **NeuN** | Millipore | Cat. # mab377 |
| **pAMPK (IF staining)** | Abcam | Cat. # ab23875 |
| **pAMPK (Western Blot)** | Cell Signaling | Cat. # 9957 |
| **TAMPK** | Abcam | Cat. # ab3760 |
| **pGSK** | Cell Signaling | Cat. # 9323S |
| **TGSK** | Cell Signaling | Cat. #9315 |
| **pTSC2** | Cell signaling | Cat. #5584S |
| **TSC2** | Cell signaling | Cat. #4308S |
| **pP70S6K (IF staining)** | Millipore | Cat. # MABS82 |
| **pP70S6K (Western Blot)** | Cell Signaling | Cat. # 9234S |
| **TP70S6K (Western Blot)** | Cell Signaling | Cat. # 2708S |
| **pPDH** | Abcam | Cat. # ab92696 |
| **TPDH** | Abcam | Cat. # ab168379 |
| **PDK4** | Thermo Fisher Scientific | Cat. # PA513776 |
| **PKM2** | Cell signaling | Cat. # 4053S |

## Software and Algorithms:

| **Reagent** | **Source** | **Identifier** |
| --- | --- | --- |
| **Zen Blue** | Carl Zeiss, Inc. | <https://www.zeiss.com/microscopy/us/downloads.html> |
| **Fiji** | Fiji | <https://imagej.net/Fiji/Downloads> |
| **Graphpad** | Prism 9.0 | https://www.graphpad.com/scientific-software/prism/ |
|  | **Chenomx 8.0** | www.chenomx.com |
|  | **Metaboanalyst 4.0** | www.metaboanalyst.ca |
