# Supplementary material for: A Warburg-like metabolic program coordinates Wnt, AMPK, and mTOR signaling pathways in epileptogenesis
Source: PLoS One. 2021 Aug 6;16(8):e0252282. doi: 10.1371/journal.pone.0252282 (PMC8345866; doi:10.1371/journal.pone.0252282)
Supplement: Attachment — Submitted filename: Response to Reviewers .docx [file pone.0252282.s012.docx]

**Author’s response to Journal Comments**

**Journal.** Please include the following items when submitting your revised manuscript:

- A rebuttal letter that responds to each point raised by the academic editor and reviewer(s). You should upload this letter as a separate file labeled 'Response to Reviewers'.
- A marked-up copy of your manuscript that highlights changes made to the original version. You should upload this as a separate file labeled 'Revised Manuscript with Track Changes'.
- An unmarked version of your revised paper without tracked changes. You should upload this as a separate file labeled 'Manuscript'.

**Authors’ Response.** Separate files of a cover letter, point-by-point rebuttal, a marked up copy, and original copy have been uploaded.

**Journal.** If you would like to make changes to your financial disclosure, please include your updated statement in your cover letter. Guidelines for resubmitting your figure files are available below the reviewer comments at the end of this letter. If applicable, we recommend that you deposit your laboratory protocols in protocols.io to enhance the reproducibility of your results. Protocols.io assigns your protocol its own identifier (DOI) so that it can be cited independently in the future. For instructions see: <http://journals.plos.org/plosone/s/submission-guidelines#loc-laboratory-protocols>

**Authors’ Response.** There are no changes in the financial disclosures.

**Journal. When submitting your revision, we need you to address these additional requirements.**

1. Please ensure that your manuscript meets PLOS ONE's style requirements, including those for file naming. The PLOS ONE style templates can be found at

<https://journals.plos.org/plosone/s/file?id=wjVg/PLOSOne_formatting_sample_main_body.pdf> and

<https://journals.plos.org/plosone/s/file?id=ba62/PLOSOne_formatting_sample_title_authors_affiliations.pdf>

**Authors’ Response.** We have used this a guide for revision.

2.  PLOS ONE now requires that authors provide the original uncropped and unadjusted images underlying all blot or gel results reported in a submission’s figures or Supporting Information files. This policy and the journal’s other requirements for blot/gel reporting and figure preparation are described in detail at <https://journals.plos.org/plosone/s/figures#loc-blot-and-gel-reporting-requirements> and <https://journals.plos.org/plosone/s/figures#loc-preparing-figures-from-image-files>. When you submit your revised manuscript, please ensure that your figures adhere fully to these guidelines and provide the original underlying images for all blot or gel data reported in your submission. See the following link for instructions on providing the original image data: <https://journals.plos.org/plosone/s/figures#loc-original-images-for-blots-and-gels>.

In your cover letter, please note whether your blot/gel image data are in Supporting Information or posted at a public data repository, provide the repository URL if relevant, and provide specific details as to which raw blot/gel images, if any, are not available. Email us at [plosone@plos.org](mailto:plosone@plos.org) if you have any questions.

**Authors’ Response.** We had included all the images of uncropped blots in the supplemental data, but have labelled them more clearly. We also consider this to be an important part of substantiating the figures.

3.  Thank you for stating the following in the Acknowledgments Section of your manuscript:

"This work was supported by grants from the Department of Defense (W81XWH-10-1-0381, ASY, ASY), the CURE Foundation (ASY and ASY) and by Umm Al-Qura University, Makkah, Saudi Arabia (RA). The work utilized NMR instrumentation that was purchased with funding from a National Institutes of Health SIG grant (S10OD020073)."

We note that you have provided funding information that is not currently declared in your Funding Statement. However, funding information should not appear in the Acknowledgments section or other areas of your manuscript. We will only publish funding information present in the Funding Statement section of the online submission form.

Please remove any funding-related text from the manuscript and let us know how you would like to update your Funding Statement. Currently, your Funding Statement reads as follows:

"ASY and ASY
Funder - Department of Defense
(W81XWH-10-1-0381)
ASY and ASY
Funder - Cure Foundation
www.cureepilepsy.org
(No Grant Number)
RA
Umm Al-Qura University, Makkah, Saudi Arabia
(Student Fellowship - no number)
The funders had no role in study design, data collection and analysis, decision to
publish, or preparation of the manuscript."

**Authors’ Response.** We have removed funding information from the acknowledgements.

This is now the correct funding information.

ASY and ASY
Funder - Department of Defense
(W81XWH-10-1-0381)
ASY and ASY
Funder - Cure Foundation
www.cureepilepsy.org
(No Grant Number)
RA
Umm Al-Qura University, Makkah, Saudi Arabia
(Student Fellowship - no number)

NMR instrumentation (National Institutes of Health SIG grant (S10OD020073)."

The funders had no role in study design, data collection and analysis, decision to
publish, or preparation of the manuscript."

4. Please amend the manuscript submission data (via Edit Submission) to include author Katherine Given.

**Authors’ response.** We have added Ms. Givens.

5. Please include captions for your Supporting Information files at the end of your manuscript, and update any in-text citations to match accordingly. Please see our Supporting Information guidelines for more information: [http://journals.plos.org/plosone/s/supporting-information](about:blank).
[Note: HTML markup is below. Please do not edit.]


 **Authors’ response.** Hopefully, the supporting information is now correctly configured.

**Author’s Response to Reviewers**

**Reviewer’s Comments to the Author:**

1. Is the manuscript technically sound, and do the data support the conclusions?

The manuscript must describe a technically sound piece of scientific research with data that supports the conclusions. Experiments must have been conducted rigorously, with appropriate controls, replication, and sample sizes. The conclusions must be drawn appropriately based on the data presented.

Reviewer #1: Yes

Reviewer #2: Partly

2. Has the statistical analysis been performed appropriately and rigorously?

Reviewer #1: I Don't Know

Reviewer #2: Yes

3. Have the authors made all data underlying the findings in their manuscript fully available?

The [PLOS Data policy](http://www.plosone.org/static/policies.action#sharing) requires authors to make all data underlying the findings described in their manuscript fully available without restriction, with rare exception (please refer to the Data Availability Statement in the manuscript PDF file). The data should be provided as part of the manuscript or its supporting information, or deposited to a public repository. For example, in addition to summary statistics, the data points behind means, medians and variance measures should be available. If there are restrictions on publicly sharing data—e.g. participant privacy or use of data from a third party—those must be specified.

Reviewer #1: No

Reviewer #2: Yes

**Authors’ Response to points 1, 2, and 3:** We have edited the manuscript to include precise descriptions of the sample sizes and underlying data to determine the statistical significance of all our findings. Throughout our studies, we emphasized statistical rigor in determining conclusions based upon our experimental data. We regret that that this very important point was not completely clear to both reviewers and have now tried to improve the clarity. All statistical analyses were performed in the latest version of Prism. The relevant calculation files are now provided in Supplemental Information. In the unrevised version, all statistical analysis was performed in Prism and statistical significance was indicated. In this revised version, we have hopefully provided sufficient details for the reviewers and readers to appreciate our rigor in analyzing the data from our many experiments.

Before submission of the first version, we had collected all the data prior to cropping for western blots and for images that were presented throughout the paper. All the blots were originally submitted as a large PDF. The revised submission now has improved labelling of these back-up files.

4. Is the manuscript presented in an intelligible fashion and written in standard English?

PLOS ONE does not copyedit accepted manuscripts, so the language in submitted articles must be clear, correct, and unambiguous. Any typographical or grammatical errors should be corrected at revision, so please note any specific errors here.

Reviewer #1: Yes

Reviewer #2: Yes

**5. Review Comments to the Author**

Please use the space provided to explain your answers to the questions above. You may also include additional comments for the author, including concerns about dual publication, research ethics, or publication ethics. (Please upload your review as an attachment if it exceeds 20,000 characters)

Reviewer #1: In their manuscript, Yee et al report that after status epilepticus (SE), an orchestrated metabolic change occurs which is governed by WNT-signaling and downstream mTOR activation, resulting in glycolysis shunting the Krebs cycle.
This paper is a very comprehensive report - commendably in many experiments based on two different SE models. Some issues, however, need to be clarified:

**Authors’ Response.** Thank you for your positive comments on the comprehensive nature of the work. This was a complex mechanism to craft to incorporate all our observations.

Major concerns:

1. Overall, there is a strong mix of methods and results, with methods often being elaborately explained in the results section or the figure legends (which were integrated in the text and often split, which made reading difficult). The authors should try to disentangle this mixture as far as possible to increase legibility.

2. Similarly, also results and introduction are mixed to some degree: Lines 110-112 and again 123-126 are somewhat summing up the results within the Introduction section. Again, this should be avoided.

3. The discussion is too long and should be condensed.

**Authors’ response to major concerns 1, 2 and 3.** We have hopefully revised the methods, results and discussion to improve readability. Because of new experiments (detailed in Figure 7) and our own realization of the elevated significance of the work, the results and discussion sections have been extensively revised, especially the latter. We attempted to shorten the discussion, but then needed to better highlight the metabolic implications. Because we added a genetic animal model of Wnt signaling (see below), we discovered that the Wnt signaling triggered role of PDH regulation may play a larger than expected role in epileptogenesis and could be part of general mechanisms, whether it be genetic or acquired.

4. Regarding Wnt and SE, the findings of Gorter et al (214, in Neurobiology of Disease No. 62) should be acknowledged.

**Authors’ response.** We have now noted this reference. Thank you.

Minor issues:

Line 45: the sentence is a bit cryptic. Please rephrase.
Line 448: Data is plural, hence please use plural also in the verb.

**Authors’ response.** We have now corrected these sentences. Yes, we did know that “data” is a plural noun, so this escaped our grammar and typo check.

Reviewer #2: The authors demonstrate a transient activation of the Wnt-beta catenin pathway during the latent period following SE similar as found in some cancers. This line of research requires investigation but the results as presented are associative and the authors do not show causality. My comments are as follow:

**Authors’ response.** Thank you for your critical and important comments. Please see below for my detailed response.

a. I would suggest toning down statements such as saying this is the first to show the complex signaling and metabolic changes underlying epileptogenesis as the authors do not show causality. Also, since a recent study by Wolff et al 2020 argues that the latent period may not even exist as subclinical seizures may continue during this period. This study potentially helps increase our understanding but without data showing that inhibition of Wnt-beta-catenin pathways leads to less spontaneous recurrent seizures following SE- these results remain an association. Especially since the changes are see only for one day. Further experiments are required to address this significant weakness of this study.

**Authors’ response.** We have revised or decreased the statements regarding the uniqueness. I’ll note that the metabolic analysis that was beautifully done by Sonnewald and others did not include this early period of epileptogenesis, but instead focused on the ~14 day period after the initiating events and during the onset and establishment of chronic seizures with the pilocarpine model in the FVB mice.

The paper by Wolff et al was very useful, as it demonstrated hyperoxia in the hippocampus within the same timeframe that we observed aerobic glycolysis in our work. Secondly, epileptiform events were centered around day 5 of epileptogenesis, also consistent with our observations. In the Wolff study, kainate was administered directly into the brain. The pharmacokinetics and pharmacodynamics of kainate delivery are likely different than our studies, where kainate was delivered intraperitoneally. This difference complicates the meaning of any comparisons.

b. It is argued that Wnt signaling inhibitors do not cross the BBB and therefore their effect was not tested or when tested was ineffective. Why not deliver these drugs icv? Especially since 2DG was ineffective in normalizing the SE Induced changes. I think this is a critical missing part of this study.

c. To increase the impact of this study it would be necessary to treat the animals with wnt-beta-catenin inhibitors and demonstrate an effect on spontaneous recurrent seizures.

**Authors’ response.** We appreciate the reviewer’s thoughtful suggestions and agree that a direct link of Wnt signaling to the events observed in this paper is important. And we grappled with functionality or our mechanistic framework throughout this work. We attempted to use of Wnt pathway inhibitors (by intraperitoneal injection) as the simplest experimental path. We used XAV939 (tankyrase inhibitor; directed at the Axin-β-catenin destruction complex) or LG974 (porcupine inhibitor; directed at synthesis of all Wnt ligands). Neither drug gave convincing results for day 5 of epileptogenesis, likely by not crossing the blood brain barrier. XAV939 has been used in a 2019 PLOS 1 study with a reported consequence to dendrite outgrowth at day 14 or so of epileptogenesis. This study used intrahippocampal injection of kainate, but intraperitoneal injection of XAV939. This study used doses of XAV939 that had been effective in an animal model of pulmonary fibrosis, which would not require brain distribution. To my knowledge, the Porcupine inhibitor has been excluded from clinical trials with brain metastases because it does not penetrate to the brain.

While the reviewer’s suggestion of using an ICV injection is an excellent idea, it is particularly difficult at this time. In this period disrupted by the covid 19 pandemic, the proposed experimental direction is extremely difficult. We have neither the equipment nor expertise to do these ICV injections. This requires a new animal protocol amendment, which at our institution now takes months for mandatory review. Furthermore, the training to do ICV injections, if we could find the equipment, is difficult due to required and necessary social distancing. Both the training and animal infrastructure at our institution has been affected by the ongoing pandemic. Furthermore, suggested approach would require multiple experiments for determination of possible brain toxicity and statistical reproducibility since there is no precedence for XAV939 or LG974 to be delivered in this manner. Our study is amongst the first studies to ponder the role of Wnt signaling in epileptogenesis, so we do not know of prior studies to guide a dose-response, even if we could learn the technical aspects of a stereotactic injections. While this would be a good proof-of principle experiment for the future, ICV would not be the mode of delivery in human patients. We are currently investigating brain-penetrant therapeutic combinations aimed at blocking Wnt signaling, but these results will be ready for publication in the not too distant future.

But the reviewers’ question is a good one. In attempting to discern whether Wnt signaling has a causal role in epileptogenesis, we took a more difficult genetic approach in the new figure 7, generated by a re-examination of some completed, albeit unpublished findings. We thank the reviewer for prompting a re-interpretation of near-completed work. The collective interpretation is that constitutive Wnt signaling increases basal metabolic enzymes and mTOR. Furthermore, constitutive Wnt signaling should decrease the threshold for susceptibility to induced seizures, and in fact does so. In the end, the new (old) findings advance the role of Wnt signaling in epileptogenesis using the mechanisms advanced in this manuscript.

Some background is necessary to interpret the new findings. To address the role of Wnt signaling in the biochemical and seizure changes, we used a mouse in which the HBP1 gene was deleted. We and others have shown that HBP1 is a transcriptional repressor that inhibits Wnt signaling. The mechanism is an inhibition of LEF/TCF transcriptional regulation (which is triggered by the upstream Wnt signaling pathway). We and others have shown that reduced levels of the HBP1 gene are associated with a poor prognosis in breast and other cancers. Because of the cancer correlation with Wnt signaling and HBP1, we sought to create a deletion of the HBP1 gene. Because of the known effect of mouse backgrounds on tumors, we carefully created strains in defined backgrounds C57BL6 and FVBN for future tumor work. These reagents are part of my lab work and were intended for our breast cancer studies. The disappointing result for the cancer studies was that the HBP1 knockouts had no spontaneous mammary tumors (though they exhibited colon cancers).

Nonetheless, a surprising observation for this paper was that these HBP1^-/-^ mice had spontaneous seizures, discovered serendipitously by our animal facility. The work of the present manuscript also showed that HBP1 decreased in early epileptogenesis, coinciding with a peak in Wnt signaling. A deletion of HBP1 exhibited increased constitutive Wnt signaling in several tissues, as would be expected by the loss of an inhibitor. Only one previous report had linked HBP1 to brain cortical differentiation. Thus we re-examined some published observations and added new experiments to create a new Figure 7. Using a genetic argument and tools, the collective conclusions of Figure 7 are that HBP1 and Wnt signaling may be part of mechanisms that set the threshold for acquired seizure susceptibility: 1) HBP1 gene expression occurs in the CA1 and DG region known to associated with regulation of seizures; 2) The deletion of HBP1 in the hippocampus results in increased Wnt signaling, mTOR activation, and metabolic rearrangements. In particular, the induction of PDK4 and PDH phosphorylation are shared in our elaborated mechanisms and in the HBP1 deletion; 3) the HBP1^+/-^ and HBP1^-/-^ mice have heightened sensitivity to seizures induced by pilocarpine in kainate in the respective mouse backgrounds,

The similarity in mechanisms between the pilocarpine and the HBP1-/- models prompted a realization that the discovered mechanisms from Wnt signaling-to-metabolism-to mTOR may be a fundamental discovery. In revising the discussion, we focused on the central role of PDH regulation and function and reviewed the literature on the very interesting role of PDH in genetic epilepsies. By obtaining the new data in Figure 7, we were now able to elevate the significance of metabolic transitions in epilepsies. We highlight in the revised manuscript that Wnt signaling is an important input into regulating PDH, which can contribute to epileptogenic, whether biochemically or genetically.

Thus, addressing the comments of the reviewers created an improved manuscript with more general and significant conclusions. We were surprised at the similarity of mechanisms, regardless of model used, and at the very central role of regulating PDH and metabolic transitions. Thus, we express our appreciation to the reviewers for their comments and thought to improve the paper.

d. I don’t clearly see the link between the mTOR and GABA story. Please remove the GABA and glutamate results which can be part of another paper.

**Authors’ response.** Yes, we do appreciate the concern of the reviewer. We do not report a relationship between mTOR and GABA. We have revised the manuscript to be absolutely sure there that a reader would not have this mistaken impression. The change in neurotransmitter homeostasis is an important aspect of this paper and feeds directly from the anaplerotic reactions of the metabolic rearrangement. Our data is most consistent with the known decrease in GABA. We show evidence that the GABA decline results from changes in metabolic homeostasis. Our data indicates that the complex glutamine-glutamate-GABA metabolic mechanisms and altered homeostasis is derived in part from the induced Warburg effect. An unappreciated and less discussed aspect of a Warburg metabolism are the anaplerotic mechanisms known to exist at the TCA intermediate alpha-ketoglutarate-to-glutamate. By systematically investigating the enzymes that contribute to glutamine/glutamate/GABA equilibrium in the brain, we observed a re-setting to new levels at day 5 that was fully consistent with the observed decrease in GABA levels. Such a mechanism fits plausibly in this period, but it is more complex than we had expected. We have attempted to further clarify the explanation. We feel that this complex mechanism now fits functionally into this paper.

e. Methods (line 944): Please explain further what is meant by “ a minimum of 4 photographic field…per mouse). For example, how many sections does this equate to. Was there attempts at a co-localization analysis? Was the person doing the counts blinded? Also please state how thick were your sections.

**Authors’ response.** We have clarified the quantitation on the figure legends and methods. From the revised methods

*Confocal Imaging:* Stained sections were imaged on a LSM800 laser scanning confocal microscope (ZEN 2.3-blue edition) in multi-tracking mode. The images were obtained with optimal pinhole a 63X, 1.4 NA, plan-apochromat oil immersion objective, 0.8 micron per optical sections, at a scan speed of 3, averaging of 4 images with an overall pixel dwell time of ~12 µsec. All image processing and analysis was performed in Fiji. In all images, only balance, contrast, and evenness of the illumination were adjusted. Final immunolabeling quantification and co-localization results were reported as percentage of positive cells (neuronal or astrocytes)/ total cells (neuronal or astrocytes). The quantification was conducted by three blinded investigators in four-six hippocampal coronal sections (section width= 12 µm) per mouse. Then, a minimum of 4 photographic fields were quantified per section (n = 3-4 mice for each group) using Fiji.

f. Methods Confocal (lines 942 and 945): Fiji and Image J are the same programs except the former has many already installed plugins.

**Authors’ response.** We apologize for the confusion. Fiji was used in all confocal images used in the figures.

g. Abstract: The following is repetitive and can be shortened “The results of this paper build a new molecular framework of complex signaling pathways for early epileptogenesis. Our framework advances the understanding of the complex molecular changes in early epileptogenesis and creates a detailed platform for discovering new future therapeutic strategies based upon Wnt signaling and/or metabolic interventions. Our studies may define a unique window in early epileptogenesis for attenuating recurrent and chronic seizures that define epilepsies.”

**Authors’ response.** We have re-written the abstract to make it more readable and to incorporate the new genetic angle. The use of two different experimental systems that yielded similar results additionally allowed some conclusions to be strengthened.

h. Introduction: Rapamycin also reduced seizures in a model of infantile spasms. Please add reference.

**Authors’ response.** Thank you. The reference has been added.

i. Introduction: May be useful to add figure which summarizes the relationship between Wnt signaling and mTOR.

**Authors’ response.** We have created a new Figure 1A to clarify the mechanisms.

j. Introduction: Frizzled receptors are mentioned without context.

**Authors’ response.** The text has been altered accordingly.

k. There are many abbreviations that require definition. Please provide a definition at their first appearance.

**Authors’ response. We have revised the manuscript to insure that abbreviations are defined.**

l. Introduction (line 114). Warburg metabolism is mentioned without a definition.

m. Introduction: (lines 115-116) …should say “that occurs in neurons” and not “…are occurs…”

**Authors’ response**. Thank you. We have revised the manuscript accordingly.

6. PLOS authors have the option to publish the peer review history of their article ([what does this mean?](https://journals.plos.org/plosone/s/editorial-and-peer-review-process#loc-peer-review-history)). If published, this will include your full peer review and any attached files.


If you choose “no”, your identity will remain anonymous but your review may still be made public.


**Do you want your identity to be public for this peer review?** For information about this choice, including consent withdrawal, please see our [Privacy Policy](https://www.plos.org/privacy-policy).

Reviewer #1: No

Reviewer #2: No
